# Causal Inference With Survey Data: A Robust Framework for Propensity Score Weighting in Probability and Non‐Probability Samples

**DOI:** 10.1002/sim.70420

**Published:** 2026-02-04

**Authors:** Wei Liang, Changbao Wu

**Affiliations:** ^1^ Department of Statistics and Actuarial Science University of Waterloo Waterloo Ontario Canada

**Keywords:** causal inference, data integration, doubly robust, non‐probability samples, observational studies, propensity score weighting, survey data

## Abstract

Confounding bias and selection bias are two major challenges in causal inference with observational data. While numerous methods have been developed to mitigate confounding bias, they often assume that the data are representative of the study population and ignore the potential selection bias introduced during data collection. In this paper, we propose a unified weighting framework—survey‐weighted propensity score weighting—to simultaneously address both confounding and selection biases when the observational dataset is a probability survey sample from a finite population, which is itself viewed as a random sample from the target superpopulation. The proposed method yields a doubly robust inferential procedure for a class of population weighted average treatment effects. We further extend our results to non‐probability observational data when the sampling mechanism is unknown but auxiliary information of the confounding variables is available from an external probability sample. We focus on practically important scenarios where the confounders are only partially observed in the external data. Our analysis reveals that the key variables in the external data are those related to both treatment effect heterogeneity and the selection mechanism. We also discuss how to combine auxiliary information from multiple reference probability samples. Monte Carlo simulations and an application to a real‐world non‐probability observational dataset demonstrate the superiority of our proposed methods over standard propensity score weighting approaches.

## 1 Introduction

Confounding bias and selection bias are two major issues in observational studies, both posing threats to causal inference with observational data: confounding undermines internal validity, while selection bias jeopardizes external validity. Confounding bias arises when baseline covariates are imbalanced between treatment groups, assuming no unmeasured confounders. However, achieving covariate balance within the sample does not guarantee results generalizable to a broader target population, as selection bias introduced by the sampling procedure may lead to covariate imbalance between the sample and the population, still distorting population‐level causal conclusions. Haneuse [1] provides a detailed discussion on confounding and selection bias, and how they are respectively related to internal and external validity in causal inference.

Propensity score weighting has been introduced as one of the major methodologies in causal inference and unconfounded association analysis for estimating average treatment effects, which mitigates confounding bias by improving distribution balance of baseline covariates between treatment groups [2]. This method offers several advantages, including ease of implementation, robustness to misspecification in outcome regression models as compared to regression‐based prediction methods, and the capacity for model checking through the assessment of covariate balance after weighting. We refer readers to Hirano et al. [3], Austin and Stuart [4], and Li et al. [5] for excellent reviews of the propensity score weighting approach and its appealing properties.

The issue of selection bias resulting from unequal probability sampling or self‐selection has received much less attention in observational studies for causal inference. Selection bias may cause a distribution shift between a sample and the target population, and its presence would lead to sample‐based evidence not directly applicable to the population. Lack of generalizability is considered to be a main concern in randomized clinical trials because of their strict inclusion criteria, which give rise to limited diversity of the candidates and the issue of extrapolation [6, 7, 8]. Observational studies, on the other hand, have the capabilities to encompass a broader and more diverse range of participants. However, this does not automatically lead to generalizability and external validity [9], especially when the characteristics associated with treatment effect heterogeneity of an observational sample are significantly different from those of the population of interest. For example, when studying the effectiveness of a drug, clinicians may be interested in the entire US population aged 18 and older, rather than the study sample alone. The population causal effect of the drug would be overestimated based on the sample if the drug is more effective among older individuals who make up a larger proportion of the sample.

In this paper, we consider two types of observational data for causal inference. We first consider observational data which are a probability survey sample under unequal probability sampling designs with known survey weights. DuGoff et al. [10] showed that while many observational datasets are collected based on a probability sampling design, a substantial portion of existing research ignores the sampling design information when doing propensity score analysis. As a result, their causal conclusions are only internally valid for the study sample but lack external validity with respect to the broader population from which the sample was drawn. Our first contribution addresses this issue by introducing a framework called “survey‐weighted propensity score weighting,” which incorporates survey weights into propensity score weighting to justify both internal and external validity of causal inference. Our theoretical results are developed under a two‐phase randomization model: The survey data are collected from a finite population, which can be viewed as a random sample from a superpopulation. Despite an increasing research interest in causal inference with complex survey data [10, 11, 12, 13, 14, 15, 16], existing literature on this problem does not fully recognize the two‐phase data generating procedure, and the role of the sampling designs in causal inference has not been rigorously addressed. Our results suggest that we should focus on evaluating the design‐based variation for inference when the finite population size is much bigger than the sample size. Under the above framework, we propose to estimate the propensity score by survey‐weighted covariate balancing approach, which yields doubly robust statistical inferential procedures for a class of population weighted average treatment effects.

A highlighted second contribution of this paper is the extension of the proposed framework to cover scenarios where the observational data are nonprobability samples. In practice, survey samples can be voluntary or collected based on non‐probability sampling methods such as judgment sampling and quota sampling [17], in which cases both the sampling mechanism and survey weights are unknown. Population‐level causal inference using non‐probability observational data is of both theoretical and practical importance for researchers to better understand causality. The problem is also more challenging due to the unknown participation mechanism. To the best of our knowledge, the topic has not been systematically studied in the existing literature. Our paper aims to provide some insights into this critical issue and develops practically useful methods for estimation of causal parameters.

Statistical analysis with non‐probability samples is an emerging topic in survey sampling and official statistics, with a focus on the estimation of population means or totals. A popular setting for methodological development in recent years is to assume the availability of a single existing reference probability sample from the same target population with the required auxiliary information [18, 19, 20, 21]. Similar data integration problems have also been explored for generalizing trial evidence. Existing methods include inverse probability weighting [22, 23, 24], the model‐assisted approach of Dahabreh et al. [25], and the non‐parametric calibration weighting method of Lee et al. [26]. We adopt this popular setting for causal inference with non‐probability observational data, assuming the information of confounding variables is available in external reference probability survey samples. In particular, we consider a practically important scenario where the confounding variables are only partially observed in the reference data. Our analysis reveals that the key variables for removing selection bias in causal inference are those who are related to both treatment effect heterogeneity and the selection mechanism. These variables can be preliminarily identified by including treatment‐by‐covariate interaction terms in the regression outcome models. The topic is further illustrated in the real data application in Section 6. In scenarios where no single existing reference probability sample provides the required auxiliary information, we develop valid inferential procedures for combining auxiliary information from two or multiple existing reference probability samples.

The rest of the paper is organized as follows: In Section 2, we describe the setting, the inferential problems of interest and notation. The proposed methodology for probability samples is presented in Section 3, with point estimation detailed in Section 3.1 and 3.2 and general inferential procedures given in Section 3.3. Extensions of the methods to non‐probability samples are described in Section 4, where the identification assumptions and statistical inference of parameters of interest are studied. Simulation studies on the performance of the proposed methods and an example of a real data application are presented respectively in Section 5 and Section 6. Some brief discussions are given in Section 7. Proofs and technical details are provided in the Supporting Information.

## 2 Setup and Notation

Let T∈{0,1} be the indicator of a treatment and suppose we are interested in the causal effect on a binary or continuous outcome Y. The Rubin causal model (RCM) is adopted [27], where Y(1) and Y(0) are defined as the two potential outcomes for Y under respectively T=1 and T=0, and the observed outcome satisfies Y=Y(T) under the Stable Unit Treatment Value Assumption [28]. Let X be a p‐dimensional vector of baseline covariates with the support 𝒳. Denote by τ(x)=E[Y(1)−Y(0)|X=x] the conditional average treatment effect (CATE). We further impose the ignorability assumption, that is, {Y(1),Y(0)}⊥T∣X, under which we have $\tau \left(x\right)=m_{1}\left(x\right)-m_{0}\left(x\right)$ where $m_{t}\left(x\right)=E\left(Y|X=x,T=t\right)$ is the conditional mean of Y for the group T=t.

Denote by e(x)=P(T=1|X=x) the propensity score (PS) function and assume that 0<e(x)<1 for x∈𝒳. We are interested in statistical inference of the population weighted average treatment effect [3] (PWATE), $\tau_{g}=E\left[g\left(X\right)\left\{Y\left(1\right)-Y\left(0\right)\right\}\right]/E\left[g\left(X\right)\right]$, where g(x) is a pre‐specified function characterizing a sub‐population of interest. Define $1_{ℬ}\left(x\right)$ as an indicator function taking the value 1 if x∈ℬ and 0 otherwise, where ℬ denotes a known subspace of 𝒳. The g(x) can be a function of the PS and $1_{ℬ}\left(x\right)$. Examples of g(x) and their corresponding causal estimands are presented in Table 1. Notably, the population estimands can be regarded as a special case of the population‐subgroup estimands when g(x)≡1. The target populations that these estimands correspond to have been discussed in Li et al. [5]. Hereafter, for simplicity, we use PSATE to refer to both PATE and PSATE when it does not cause any confusion; the same convention applies to PSATT, PSATC, and PSATO. In this paper, we focus on the estimation of the PSATE, PSATT, and PSATO, which have received considerable attention in the causal inference literature.

**TABLE 1:** Examples of population and population‐subgroup estimands under different g(x).

g(x)	Estimand
1	Population average treatment effect (PATE)
e(x)	Population average treatment effect for the treated (PATT)
1−e(x)	Population average treatment effect for the control (PATC)
e(x)(1−e(x))	Population average treatment effect for the overlap (PATO)
$1_{ℬ}\left(x\right)$	Population‐subgroup average treatment effect (PSATE)
$1_{ℬ}\left(x\right)e\left(x\right)$	Population‐subgroup average treatment effect for the treated (PSATT)
$1_{ℬ}\left(x\right)\left(1-e\left(x\right)\right)$	Population‐subgroup average treatment effect for the control (PSATC)
$1_{ℬ}\left(x\right)e\left(x\right)\left(1-e\left(x\right)\right)$	Population‐subgroup average treatment effect for the overlap (PSATO)

Consider a finite population with N labeled units, ${𝒰}_{N}=\left\{1,\cdots ,N\right\}$, and a probability survey sample ${𝒮}_{A}\subset {𝒰}_{N}$ drawn from the finite population based on a probability sampling design, denoted by q. The observed data are $\left\{\left(Y_{i},T_{i},X_{i},d_{i}^{A}\right),i\in {𝒮}_{A}\right\}$ where $d_{i}^{A}={\left\{\pi_{i}^{A}\right\}}^{-1}$ are survey weights and $\pi_{i}^{A}=P_{q}\left(i\in {𝒮}_{A}\right)$ are first‐order inclusion probabilities. We use n to denote the size of ${𝒮}_{A}$.

Regarding the temporal ordering of treatment assignment and survey sampling, observational survey data can generally be classified into two types: pre‐treatment observational data, in which treatment is assigned after the sampling units are recruited, and post‐treatment observational data, in which treatment assignment occurs prior to survey sample selection. In the context of probability survey samples, post‐treatment observational data are more common when the treatment indicators are observed rather than assigned. Therefore, we focus primarily on post‐treatment observational data in this paper. Discussions of pre‐treatment observational data in the context of non‐probability samples are given in Section 4.5.

To simplify notation, let O=(Y,T,X) and $O_{i}=\left(Y_{i},T_{i},X_{i}\right)$. We assume that $\left\{O_{i},i\in {𝒰}_{N}\right\}$ are N independent and identically distributed copies of O, that is, the finite population is viewed as a random sample from the superpopulation, and the observed data are a probability survey sample from the finite population. This kind of two‐phase joint randomization has been widely accepted in survey sampling as a framework for model‐based and model‐assisted inference [17, 29, 30]. The framework is adopted in our current context of super‐population causal inference and finite‐population survey sampling. It is crucial to note that randomness for the design q solely comes from the probability sampling procedure. We use $E_{q}\left(\cdot \right)$ and $V_{q}\left(\cdot \right)$ to denote the expectation and variance under the design q, and E(⋅) and V(⋅) for the expectation and variance under the joint model. Note that the sample size n could be a random number under certain sampling designs. For any function u(⋅) of O, we assume the sampling design q and the finite population satisfy

- $d_{i}^{A}=O_{p}\left(N/n\right)$ and $\pi_{i}^{A}=O_{p}\left(n/N\right)$;
- $E_{q}\left[\sum_{i\in {𝒮}_{A}}d_{i}^{A}u\left(O_{i}\right)\right]=\sum_{i=1}^{N}u\left(O_{i}\right);$
- $N^{-1}\sum_{i\in {𝒮}_{A}}d_{i}^{A}u\left(O_{i}\right)=N^{-1}\sum_{i=1}^{N}u\left(O_{i}\right)+o_{p}\left(1\right)$ if $E\left[{\left\{u\left(O\right)\right\}}^{2}\right]<\infty$.

The three assumptions are commonly used in the survey sampling literature [17].

The asymptotic framework for the two‐phase joint model is as follows. Let $\left\{{𝒰}_{v},v=1,\cdots \right\}$ be a sequence of finite populations with the population size $\left\{N_{v},v=1,\cdots \right\}$; let ${𝒮}_{v}$ be a sample of size $n_{v}$ drawn from ${𝒰}_{v}$. As v→∞, $N_{v}\to \infty$ and $n_{v}\to \infty$. For ease of notation, we suppress the subscript v and use N→∞ to denote the limiting process.

## 3 Survey‐Weighted Propensity Score Weighting

Propensity score weighting (PSW) is often employed in observational studies to mitigate confounding bias arising from nonrandomized treatment assignments for causal inference [5]. Naive application of PSW to survey data, however, may result in biased estimation and invalid conclusions on the target population due to potential selection bias; see the toy example presented in the Supporting Information for illustration. We combine inverse PS weights with survey weights to simultaneously address the issue of confounding and selection bias.

Let us postulate a parametric model, $e\left(x\right)=e_{\beta }\left(x\right)$, for the PS and assume the parameter β lies in a compact set $\Theta_{\beta }$. We consider the following weight

$$\omega_{i}\left(\beta \right)=d_{i}^{A}\left\{\frac{T_{i}g\left(X_{i}\right)}{e_{\beta }\left(X_{i}\right)}+\frac{\left(1-T_{i}\right)g\left(X_{i}\right)}{1-e_{\beta }\left(X_{i}\right)}\right\}$$

for the sample unit i where the function g(x), if defined in terms of the PS, also uses the postulated parametric form $e_{\beta }\left(x\right)$. The inverse PS weights balance the distribution of baseline covariates between the treated and control groups and the survey weights make the sample representative of the finite population. Let $\beta_{0}\in \Theta_{\beta }$ be the true value of β when the PS model is correctly specified such that $e\left(x\right)=e_{\beta_{0}}\left(x\right)$, we have

$$\begin{array}{cc} & E\left[\frac{1}{N}\sum_{i\in {𝒮}_{A}}\omega_{i}\left(\beta_{0}\right)\left(2T_{i}-1\right)Y_{i}\right]=E\left[\frac{1}{N}\sum_{i=1}^{N}\left\{\frac{T_{i}g\left(X_{i}\right)}{e_{\beta_{0}}\left(X_{i}\right)}-\frac{\left(1-T_{i}\right)g\left(X_{i}\right)}{1-e_{\beta_{0}}\left(X_{i}\right)}\right\}Y_{i}\right]\\ & \;=E\left[\tau \left(X\right)g\left(X\right)\right]. \end{array}$$

This formulation suggests the following Hájek‐type estimator for $\tau_{g}$,

(1) $$\hat{\tau }=\frac{\sum_{i\in {𝒮}_{A}}T_{i}{\hat{\omega }}_{i}Y_{i}}{\sum_{i\in {𝒮}_{A}}T_{i}{\hat{\omega }}_{i}}-\frac{\sum_{i\in {𝒮}_{A}}\left(1-T_{i}\right){\hat{\omega }}_{i}Y_{i}}{\sum_{i\in {𝒮}_{A}}\left(1-T_{i}\right){\hat{\omega }}_{i}},$$

where ${\hat{\omega }}_{i}=\omega_{i}\left(\hat{\beta }\right)$ and $\hat{\beta }$ is an estimator of β. This will be called survey‐weighted inverse probability weighting (SW‐IPW) estimator. We discuss how to estimate β under the current setting in Sections 3.1 and 3.2. The weights $\omega_{i}\left(\beta \right)$ can also be combined with regression outcome models for causal inference [31, 32].

### 3.1 Estimation of Propensity Score

If we observe the entire finite‐population data, we can estimate β by maximizing the log‐likelihood

$$\ell_{N}\left(\beta \right)=\sum_{i=1}^{N}T_{i}\log e_{\beta }\left(X_{i}\right)+\sum_{i=1}^{N}\left(1-T_{i}\right)\log\left\{1-e_{\beta }\left(X_{i}\right)\right\}.$$

Using the sample data with known survey weights to approximate $\ell_{N}\left(\beta \right)$, we propose to estimate $\beta_{0}$ by maximizing the pseudo log‐likelihood

(2) $$\ell_{n}\left(\beta \right)=\sum_{i\in {𝒮}_{A}}d_{i}^{A}T_{i}\log e_{\beta }\left(X_{i}\right)+\sum_{i\in {𝒮}_{A}}d_{i}^{A}\left(1-T_{i}\right)\log\left\{1-e_{\beta }\left(X_{i}\right)\right\},$$

or equivalently, solving the pseudo score equations for β as

(3) $$0=\frac{\partial \ell_{n}\left(\beta \right)}{\partial \beta }=\sum_{i\in {𝒮}_{A}}d_{i}^{A}h_{\mathrm{BL}}\left(X_{i},T_{i},\beta \right),$$

where

$$h_{\mathrm{BL}}\left(X_{i},T_{i},\beta \right)=\frac{T_{i}e_{\beta }^{\prime }\left(X_{i}\right)}{e_{\beta }\left(X_{i}\right)}-\frac{\left(1-T_{i}\right)e_{\beta }^{\prime }\left(X_{i}\right)}{1-e_{\beta }\left(X_{i}\right)}$$

and $e_{\beta }^{\prime }\left(X_{i}\right)$ is the first derivative of $e_{\beta }\left(X_{i}\right)$ with respect to β. When the PS model is correctly specified, the maximum pseudo likelihood estimation (MPLE) approach yields consistent estimator for $\beta_{0}$ under suitable regularity conditions on the smoothness [33].

More generally, let Q(β)=E[h(X,T,β)] and $Q_{n}\left(\beta \right)=N^{-1}\sum_{i\in {𝒮}_{A}}d_{i}^{A}h\left(X_{i},T_{i},\beta \right)$ for some vector function h. If every component of h has finite second moment at each $\beta \in \Theta_{\beta }$, then we have $Q_{n}\left(\beta \right)=N^{-1}\sum_{i=1}^{N}h\left(X_{i},T_{i},\beta \right)+o_{p}\left(1\right)=Q\left(\beta \right)+o_{p}\left(1\right)$ for each $\beta \in \Theta_{\beta }$ by the weak law of large numbers. Therefore, we expect the solution to $Q_{n}\left(\beta \right)=0$ also converges to the solution to Q(β)=0. However, as pointed out by Vaart [34], the pointwise convergence is too weak to guarantee consistency of the estimator, for which we need stronger assumptions.

Theorem 1 *Suppose* $\beta_{0}$ *is the unique solution to* Q(β)=0 *over* $\Theta_{\beta }$ *and* $\hat{\beta }$ *is a solution to* $Q_{n}\left(\beta \right)=0$. *Then, we have* $\hat{\beta }=\beta_{0}+o_{p}\left(1\right)$ *if* Q(β) *is continuous in* β *and* $Q_{n}\left(\beta \right)$ *converges in probability and uniformly in* $\beta \in \Theta_{\beta }$ *to* Q(β). *The uniform convergence condition can be weakened to pointwise convergence if* $Q_{n}\left(\beta \right)$ *is continuous and the solution to* $Q_{n}\left(\beta \right)=0$ *over* $\Theta_{\beta }$ *is also unique*.

The proposition of Theorem 1 follows directly from theorem 5.9 and lemma 5.10 in Vaart [34]. The key to obtaining a consistent estimator of $\beta_{0}$ is that we need to find an unbiased estimating function for $\beta_{0}$ and solve the corresponding survey‐weighted estimating equations. An alternative formulation of the unbiased survey‐weighted estimating Equation (3), where $e_{\beta }^{\prime }\left(X_{i}\right)$ is replaced by $g\left(X_{i}\right)X_{i}$, is discussed in Section 3.2. With a consistent estimator for $\beta_{0}$, we can verify that $\hat{\tau }$ is also consistent for $\tau_{g}$. The proof is provided in the Supporting Information.

Theorem 2 *When the PS model is correctly specified, assume that* $e_{\beta }\left(X\right)$ *is continuous in* β *almost surely in a neighborhood of* $\beta_{0}$. *Then, for any* $\hat{\beta }=\beta_{0}+o_{p}\left(1\right)$, *the resulting SW‐IPW estimator in* (1) *is consistent for* $\tau_{g}$ *if* $E\left(Y^{2}|T=t\right)<\infty$ *for* t=0,1.

While it is natural to incorporate the survey weights when re‐weighting the outcomes, it may not be immediately clear to some readers whether the survey weights are also necessary for estimating the PS. To clarify this point, suppose the survey weights are ignored in the estimation of β. Then, standard approaches for estimating the PS identifies the local PS, $e_{l}\left(X_{i}\right)=P\left(T_{i}=1|X_{i},i\in {𝒮}_{A}\right)$. If $\tau_{g}$ is defined in terms of e(X) such as the PSATT and PSATO, it is clear that the survey weights are essential unless $e\left(X_{i}\right)=e_{l}\left(X_{i}\right)$, which is violated when the inclusion probability $\pi_{i}^{A}$ is dependent of $T_{i}$ given $X_{i}$. This is closely related to non‐ignorable missing mechanism in missing data problems [35]; see the Supporting Information for further illustration. The survey weights are also needed for estimating the PSATE. Without loss of generality, consider the estimation of $\tau_{g,1}=E\left[Y\left(1\right)\right]$ and define the weights $\omega_{i}^{*}=d_{i}^{A}\left\{T_{i}/e_{l}\left(X_{i}\right)+\left(1-T_{i}\right)/\left(1-e_{l}\left(X_{i}\right)\right)\right\}$. It can be seen that

$$\frac{E\left[\sum_{i\in {𝒮}_{A}}\omega_{i}^{*}T_{i}Y_{i}\right]}{E\left[\sum_{i\in {𝒮}_{A}}\omega_{i}^{*}T_{i}\right]}=\frac{E\left[Y_{i}\left(1\right)e\left(X_{i}\right)/e_{l}\left(X_{i}\right)\right]}{E\left[e\left(X_{i}\right)/e_{l}\left(X_{i}\right)\right]},$$

which is not equal to E[Y(1)] unless $e\left(X_{i}\right)/e_{l}\left(X_{i}\right)$ is independent of $Y_{i}\left(1\right)$. Even when $e\left(X\right)=e_{l}\left(X\right)$, we still encourage the use of the survey weights in the estimation of the PS. See Section 3.2 for further discussion.

### 3.2 Survey‐Weighted Covariate Balancing Propensity Score Weighting

Covariate balancing propensity score (CBPS) method for the estimation of β was initially proposed by Imai and Ratkovic [36] as solving over‐identified estimating equations involving both score functions and a covariate balancing condition. An alternative form of the CBPS method is to solve a set of just‐identified equations that only contains the covariate balancing condition. Suppose the constant 1 has been included in the covariates. Under a logistic regression model for the PS, we have $e_{\beta }\left(x\right)=\text{expit}\left(\beta^{⊤}x\right)$, where expit(z)=1/{1+exp(−z)}. Define the covariate balancing functions as

$$h_{\mathrm{CB},g}\left(X_{i},T_{i},\beta \right)=\left\{\frac{T_{i}g\left(X_{i}\right)}{e_{\beta }\left(X_{i}\right)}-\frac{\left(1-T_{i}\right)g\left(X_{i}\right)}{1-e_{\beta }\left(X_{i}\right)}\right\}X_{i},$$

which is a set of unbiased estimating functions for $\beta_{0}$ when the PS model is correctly specified. We propose to estimate β by solving $\sum_{i\in {𝒮}_{A}}d_{i}^{A}h_{\mathrm{CB},g}\left(X_{i},T_{i},\beta \right)=0$, termed as the survey‐weighted CBPS (SW‐CBPS) approach.

The naive CBPS method produces weights that balance the covariate means between the treated and control groups, but does not yield results generalizable to the target population. By incorporating the survey weights, the resulting weights $\omega_{i}\left(\beta \right)$ not only yield covariate balance between treatment groups, but also improve covariate balance between the treatment groups and the target population. When the PS model is correctly specified, one can easily verify that $E\left[\sum_{i\in {𝒮}_{A}}T_{i}\omega_{i}\left(\beta_{0}\right)u\left(X_{i}\right)\right]=E\left[\sum_{i\in {𝒮}_{A}}\left(1-T_{i}\right)\omega_{i}\left(\beta_{0}\right)u\left(X_{i}\right)\right]=E\left[u\left(X_{i}\right)\right]$ for any function u(x).

When the PS model is wrong, the weights created by the SW‐CBPS method still balance covariate means between the treated and control groups. For ease of notation, let ${\tilde{d}}_{i}^{A}=d_{i}^{A}1_{ℬ}\left(X_{i}\right)$. Adopting the techniques introduced in Zhao [37], we can show that the weights for estimating the PSATE ($g\left(x\right)=1_{ℬ}\left(x\right)$) obtained by the SW‐CBPS method are the solutions of the following optimization problem:

(4) $$\begin{array}{cc} & \min_{\omega_{i}\geq {\tilde{d}}_{i}^{A}}\;\sum_{i\in {𝒮}_{A}}\left\{\left(\omega_{i}-{\tilde{d}}_{i}^{A}\right)\log\left(\omega_{i}-{\tilde{d}}_{i}^{A}\right)-\omega_{i}\left(1+\log\left({\tilde{d}}_{i}^{A}\right)\right)\right\}\\ & s.t.\sum_{i\in {𝒮}_{A},T_{i}=1}\omega_{i}X_{i}=\sum_{i\in {𝒮}_{A},T_{i}=0}\omega_{i}X_{i}. \end{array}$$

The dual problem formulation of (4) for estimating the PSATT is given in the Supporting Information. The objective function of (4) reaches its global minimum at $\omega_{i}={\tilde{d}}_{i}^{A}$ for $i\in {𝒮}_{A}$, implying that the estimated weights are forced towards the survey weights subject to the covariate balancing condition. It is worth noting that many popular non‐parametric calibration methods in causal inference aim to solve an optimization problem with the objective function taking a variety of forms such as the empirical likelihood function [38], cross entropy [39], and the general distance measure [40]. Unfortunately, all these methods do not incorporate the survey weights into their frameworks and simply push the estimated weights to the uniform weights rather than the survey weights. Hence, like the CBPS method, they only achieve covariate (mean) balance between the treated and the control groups. Our proposed SW‐CBPS method is expected to further improve covariate balance between the treatment groups and the population of interest. Such three‐way covariate balance leads to robust estimation of $\tau_{g}$.

Theorem 3 *(Robustness). When the PS model is wrong, the SW‐IPW estimators for the PSATE and PSATO based on the SW‐CBPS method remain consistent if* $m_{0}\left(x\right)$ *is linear in* x *and* τ(x) *is a constant. For estimating the PSATT, the constant assumption on the CATE* τ(x) *can be removed and the robustness property remains*.

When the CATE is not a constant, achieving statistical consistency under a misspecified PS model requires that the resulting weights satisfy exact three‐way covariate balance [40, 41]. However, this condition is not guaranteed by our approach because the exact three‐way covariate balancing condition

$$\sum_{i\in {𝒮}_{A}}T_{i}\omega_{i}\left(\beta \right)X_{i}=\sum_{i\in {𝒮}_{A}}\left(1-T_{i}\right)\omega_{i}\left(\beta \right)X_{i}=\sum_{i\in {𝒮}_{A}}d_{i}^{A}X_{i}$$

is an over‐identified equation system for β, which typically has no solutions. We emphasize that exact three‐way covariate balance is a sufficient condition for theoretical robustness but is not essential in our numerical experiment. The constant CATE assumption is therefore imposed solely to facilitate theoretical analysis. Also, this assumption is not necessary in theory for estimating the PSATT because the target population for estimating the PSATT only contains the treated individuals, thus a two‐way covariate balance between the treated group and the target population is sufficient.

The CBPS‐based weighting estimators are closely related to the augmented inverse probability weighting (AIPW) estimator when the regression models for the potential outcomes are linear in the observed covariates. See, for example, Zhao and Percival [42], Zhao [37] and Fan et al. [41] for further detail. It is well‐known that the AIPW estimator reaches the semiparametric efficiency bound when the PS and outcome regression models are both correctly specified [43]. Therefore, the SW‐IPW estimator based on the SW‐CBPS approach is also expected to have smaller variance than the one based on the MPLE method when the linear models fit $m_{0}\left(x\right)$ and $m_{1}\left(x\right)$ well.

### 3.3 Statistical Inference

Our inferential procedure for $\tau_{g}$ is based on the asymptotic distribution of $\hat{\tau }-\tau_{g}$ under the joint model. The problem is challenging in general, but it can be considerably simplified when the sampling fraction n/N is small, which is often the case in practice, especially when the sample data are collected from a large population. Heuristically, suppose $\tilde{\tau }$ is the limit of $\hat{\tau }$ under the design q. Then, we can decompose $n^{1/2}\left(\hat{\tau }-\tau_{g}\right)$ as

$$n^{1/2}\left(\hat{\tau }-\tau_{g}\right)=n^{1/2}\left(\hat{\tau }-\tilde{\tau }\right)+{\left(n/N\right)}^{1/2}N^{1/2}\left(\tilde{\tau }-\tau_{g}\right).$$

Under suitable regularity conditions, we have $N^{1/2}\left(\tilde{\tau }-\tau_{g}\right)=O_{p}\left(1\right)$. If we further assume that $n/N=o_{p}\left(1\right)$, then the limiting distribution of $n^{1/2}\left(\hat{\tau }-\tau_{g}\right)$ is dominated by the limiting behavior of $n^{1/2}\left(\hat{\tau }-\tilde{\tau }\right)$, which is expected to be asymptotically normal in the law of q. Hence, the critical step for inference of $\tau_{g}$ is to estimate the design‐based variance of $\hat{\tau }$. An asymptotic (1−α) confidence interval for $\tau_{g}$ can be constructed as $\left[\hat{\tau }-z_{\alpha /2}{\hat{V}}_{q}\left(\hat{\tau }\right),\hat{\tau }+z_{\alpha /2}{\hat{V}}_{q}\left(\hat{\tau }\right)\right]$ where $z_{\alpha }$ is the upper α quantile of standard normal distribution and ${\hat{V}}_{q}\left(\hat{\tau }\right)$ is a design‐consistent estimator for $V_{q}\left(\hat{\tau }\right)$.

Let $\sum_{i\in {𝒮}_{A}}d_{i}^{A}h\left(X_{i},T_{i},\beta \right)=0$ be the survey‐weighted estimating equations used to solve for β such that $E\left[h\left(X,T,\beta_{0}\right)\right]=0$ when the PS model is correctly specified. Let $\eta ={\left(\beta^{⊤},\tau_{1},\tau \right)}^{⊤}\in \Theta_{\beta }\times {\mathbb{R}}^{2}$ be the vector of parameters involved in estimation where $\tau_{1}$ is a nuisance parameter used to define the main parameter of interest, τ. The proposed procedure for estimating $\tau_{g}$ based on the SW‐IPW estimators can be formatted as solving the system of estimating equations

(5) $$0=\sum_{i\in {𝒮}_{A}}d_{i}^{A}\psi_{g,h}\left(O_{i},\eta \right),$$

where

$$\psi_{g,h}\left(O_{i},\eta \right)=\left\{\begin{array}{c} h\left(X_{i},T_{i},\beta \right)\\ \frac{T_{i}g\left(X_{i}\right)}{e_{\beta }\left(X_{i}\right)}\left(Y_{i}-\tau_{1}\right)\\ \frac{\left(1-T_{i}\right)g\left(X_{i}\right)}{1-e_{\beta }\left(X_{i}\right)}\left(Y_{i}-\tau_{1}+\tau \right) \end{array}\right\}.$$

For simplicity of notation, let $Q\left(\eta \right)=E\left[\psi_{g,h}\left(O,\eta \right)\right]$, $Q_{N}\left(\eta \right)=N^{-1}\sum_{i=1}^{N}\psi_{g,h}\left(O_{i},\eta \right)$, and $Q_{n}\left(\eta \right)=N^{-1}\sum_{i\in {𝒮}_{A}}d_{i}^{A}\psi_{g,h}\left(O_{i},\eta \right)$. Denote by $\eta^{*}$, $\tilde{\eta }$ and $\hat{\eta }$ respectively the solution to Q(η)=0, $Q_{N}\left(\eta \right)=0$, and $Q_{n}\left(\eta \right)=0$. It is expected that $\hat{\eta }=\tilde{\eta }+o_{p}\left(1\right)=\eta^{*}+o_{p}\left(1\right)$ under suitable conditions. The consistency of $\hat{\tau }$ for $\tau_{g}$ has been studied in preceding subsections, under which we have $\tau^{*}=\tau_{g}$. We now present the limiting distribution of $\hat{\tau }-\tau^{*}$ in the following theorem, which forms the basis for statistical inference.

Theorem 4 *Under Assumptions* **A1** *–* **A4** *given in the* Supporting Information, *suppose* $n^{1/2}Q_{n}\left(\tilde{\eta }\right)$ *converges in the law of* q *to* N(0,Ω) *as* N→∞ *where the asymptotic variance–covariance matrix* Ω *is finite and invertible (almost surely), and the sampling fraction* n/N *converges in probability to* 0 *as* N→∞. *Then, we have* $n^{1/2}\Omega^{-1/2}D\left(\hat{\eta }-\eta^{*}\right)$ *converges under the joint model in distribution to a standard multivariate normal distribution as* N→∞ *where* $D=E\left[\partial \psi_{g,h}\left(O,\eta^{*}\right)/\partial \eta^{⊤}\right]$.

Theorem 4 is established under the assumption that N is much bigger than n. When the sampling fraction is statistically greater than 0, deriving the asymptotic distribution of $\hat{\eta }$ under the joint model requires that the limiting distribution of $n^{1/2}\left(\hat{\eta }-\tilde{\eta }\right)$ under the design q is nonstochastic under the super‐population model; see Rubin‐Bleuer and Kratina [30] for further detail. This assumption is not required in Theorem 4.

Corollary 1 *Let* ${\hat{V}}_{n}$ *be an estimator of the design‐based theoretical covariance of* $Q_{n}\left(\tilde{\eta }\right)$ *that satisfies* ${\hat{V}}_{n}=V_{q}\left\{Q_{n}\left(\tilde{\eta }\right)\right\}+o_{p}\left(n^{-1}\right)$. *Let* ${\hat{D}}_{n}={\hat{N}}^{-1}\sum_{i\in {𝒮}_{A}}d_{i}^{A}\partial \psi_{g,h}\left(O,\hat{\eta }\right)/\partial \eta^{⊤}$ *where* $\hat{N}=\sum_{i\in {𝒮}_{A}}d_{i}^{A}$. *Let* ${\hat{\sigma }}_{n}^{2}$ *be the last diagonal element of* ${\hat{D}}_{n}^{-1}\left({\hat{V}}_{n}\right){\hat{D}}_{n}^{-1}$ *and* $C_{\alpha }=\left[\hat{\tau }-z_{\alpha /2}{\hat{\sigma }}_{n},\hat{\tau }+z_{\alpha /2}{\hat{\sigma }}_{n}\right]$. *Under the assumptions in Theorem* 4 *and when* $\tau^{*}=\tau_{g}$ *(i.e*., $\hat{\tau }=\tau_{g}+o_{p}\left(1\right)$ *), we have* $P\left(\tau_{g}\in C_{\alpha }\right)\to 1-\alpha$ *as* N→∞.

Corollary 1 follows from Theorem 4 due to Slutsky's theorem, which demonstrates that the key to valid inference of $\tau_{g}$ under our proposed framework is a consistent SW‐IPW estimator of $\tau_{g}$ and an estimator for the design‐based variance–covariance matrix of $Q_{n}\left(\tilde{\eta }\right)$, which is given by

$$V_{q}\left\{Q_{n}\left(\tilde{\eta }\right)\right\}=\frac{1}{N^{2}}\sum_{i=1}^{N}\sum_{j=1}^{N}\frac{\pi_{\mathrm{ij}}^{A}-\pi_{i}^{A}\pi_{j}^{A}}{\pi_{i}^{A}\pi_{j}^{A}}\psi_{g,h}{\left(O_{i},\tilde{\eta }\right)}^{⊗2},$$

where $\pi_{\mathrm{ij}}^{A}=P\left(i,j\in {𝒮}_{A}\right)$ is the second‐order inclusion probability for the pair (i,j); see Wu and Thompson [17] for design‐based variance formulas for survey‐weighted estimators. The variance–covariance matrix $V_{q}\left\{Q_{n}\left(\tilde{\eta }\right)\right\}$ can be consistently estimated by ${\hat{V}}_{n}={\hat{N}}^{-2}\sum_{i\in {𝒮}_{A}}\sum_{j\in {𝒮}_{A}}\left(\pi_{\mathrm{ij}}^{A}-\pi_{i}^{A}\pi_{j}^{A}\right){\left(\pi_{\mathrm{ij}}^{A}\pi_{i}^{A}\pi_{j}^{A}\right)}^{-1}\psi_{g,h}{\left(O_{i},\hat{\eta }\right)}^{⊗2}$.

The $\pi_{\mathrm{ij}}^{A}$'s may be difficult to compute under complex survey designs, and practitioners often use approximate variance estimator not involving $\pi_{\mathrm{ij}}^{A}$. For instance, one may use the variance formula under Poisson sampling,

(6) $${\hat{V}}_{n}=\frac{1}{{\hat{N}}^{2}}\sum_{i\in {𝒮}_{A}}\frac{1-\pi_{i}^{A}}{{\left(\pi_{i}^{A}\right)}^{2}}\psi_{g,h}{\left(O_{i},\hat{\eta }\right)}^{⊗2}.$$

Another example is the Hájek variance estimator which performs well for the so‐called high entropy survey designs; see section 4.2.2 of Wu and Thompson [17] for further detail. According to Corollary 1, the proposed inferential procedure remains valid as long as the SW‐IPW estimator is consistent for $\tau_{g}$. Furthermore, when β is estimated using the SW‐CBPS method, Theorem 3 ensures that the inferential procedure is also doubly robust.

## 4 Causal Inference With Non‐Probability Samples

In this section, we extend our proposed methodologies to cover scenarios where the observational dataset is a non‐probability sample from the finite population ${𝒰}_{N}$ with unknown sample selection mechanisms. Let ${𝒮}_{A}$ be the non‐probability sample and $R_{i}=1\left\{i\in {𝒮}_{A}\right\}$ be the sample inclusion indicator for unit i. We assume that the unknown sample selection mechanism is guided by an underlying probability model on R and $\left\{\left(Y_{i},T_{i},X_{i},R_{i}\right),i\in {𝒰}_{N}\right\}$ are independent and identically distributed copies of (Y,T,X,R). We use E(⋅) and V(⋅) to denote the expectation and variance under the probability distribution for (Y,T,X,R).

### 4.1 Survey‐Weighted Propensity Score Weighting Using Participation Probabilities

Perhaps the most crucial task in dealing with non‐probability samples is to estimate the so‐called participation probability π(X)=P(R=1|X). Two commonly used assumptions are (i) E(Y|X,T,R)=E(Y|X,T), where the inclusion of T is unique to our current setting; and (ii) There exists a reference probability sample, ${𝒮}_{B}$, with information on X. Wu [44] contains general discussions on statistical analysis with non‐probability samples.

We consider practical scenarios where not all components of X are observed in the reference sample ${𝒮}_{B}$. For example, the observational dataset may contain both demographic information and individual health condition measurements, whereas the external reference dataset includes only demographic variables. Let 𝒞 be the set of indices of observed components of X for ${𝒮}_{B}$ and $X_{𝒞}$ the corresponding observed covariates. The reference dataset is given by ${\left\{\left(X_{𝒞,i},d_{i}^{B}\right)\right\}}_{i\in {𝒮}_{B}}$, where $d_{i}^{B},i\in {𝒮}_{B}$ are the known survey weights for ${𝒮}_{B}$. Let $\pi \left(X_{𝒞}\right)=E\left(R|X_{𝒞}\right)$ and assume that $0<\pi \left(X_{𝒞}\right)<1$ over the support of $X_{𝒞}$. We postulate a parametric model for the participation probability, denoted by $\pi \left(X_{𝒞}\right)=\pi_{\theta }\left(X_{𝒞}\right)$, assume $\Theta_{\theta }$ is the parameter space for θ and $\theta_{0}$ is the true value when the model is correct, and focus on the following weights for the sample units:

(7) $$\omega_{i}\left(\beta ,\theta \right)=\frac{1}{\pi_{\theta }\left(X_{𝒞,i}\right)}\left\{\frac{T_{i}g\left(X_{i}\right)}{e_{\beta }\left(X_{i}\right)}+\frac{\left(1-T_{i}\right)g\left(X_{i}\right)}{1-e_{\beta }\left(X_{i}\right)}\right\}.$$

Compared to the weights $\omega_{i}\left(\beta \right)$ for probability samples, the known survey weights are replaced by inverse participation probabilities in $\omega_{i}\left(\beta ,\theta \right)$. The additional step to handle non‐probability samples is to estimate θ. The parameter β can be estimated by either the MPLE method or the SW‐CBPS method as in Section 3, where the known survey weights are replaced by the inverse of estimated participation probabilities. Once we obtain the estimators $\hat{\theta }$ and $\hat{\beta }$, we propose to estimate $\tau_{g}$ by the SW‐IPW estimator as given in (1) where ${\hat{\omega }}_{i}=\omega_{i}\left(\hat{\beta },\hat{\theta }\right)$.

With a single reference dataset with information on X, Chen et al. [21] proposed to estimate θ by maximizing the pseudo log‐likelihood

(8) $$\ell_{n}\left(\theta \right)=\sum_{i\in {𝒮}_{A}}\log\left\{\frac{\pi_{\theta }\left(X_{𝒞,i}\right)}{1-\pi_{\theta }\left(X_{𝒞,i}\right)}\right\}+\sum_{i\in {𝒮}_{B}}d_{i}^{B}\log\left\{1-\pi_{\theta }\left(X_{𝒞,i}\right)\right\}.$$

Our focus for the rest of the section is to (i) discuss identification assumptions for $\tau_{g}$; and (ii) propose a new approach for estimating θ when information on certain components of $X_{𝒞}$ is scattered in two or multiple existing reference probability samples.

### 4.2 Identification With Partially Observed Covariates

The $\theta_{0}$, $\beta_{0}$ and $\tau_{g}$ are three parameters to be identified. Identification of $\theta_{0}$ based on a single reference survey sample has been comprehensively discussed in Chen et al. [21] and Wu [44]. We focus on (i) identification $\beta_{0}$ under $\theta_{0}$ and (ii) identification of $\tau_{g}$ under $\theta_{0}$ and $\beta_{0}$. We first discuss identification assumptions for $\beta_{0}$.

Theorem 5 *Under the condition* **C1** *:* T⊥R∣X, *we have* $E\left\{\left[R/\pi_{\theta_{0}}\left(X_{𝒞}\right)\right]h\left(X,T,\beta_{0}\right)\right\}=0$ *and also* $E\left[Rh\left(X,T,\beta_{0}\right)\right]=0$, *where* $h=h_{\mathrm{BL}}$ *or* $h=h_{\mathrm{CB},g}$ *as defined in Section* 3.

Since we can only observe T for R=1, the T for R=0 can be treated as missing values and **C1** requires T is missing at random [35], under which the PS is equal to the local PS, that is, e(X)=P(T=1|R=1,X). Therefore, the participation probabilities are not essential for estimating β. However, incorporating the participation probabilities in the estimation of β can be beneficial in certain settings. For instance, achieving exact covariate mean balance between the treated and control groups based on the SW‐CBPS method requires the use of estimated participation probabilities. When adopting the MPLE approach, the decision to weight or not is less straightforward, and a direct comparison of the variances of the resulting estimators for $\tau_{g}$ is not a trivial task. In this paper, we choose to incorporate the estimated participation probabilities when estimating the PS function to maintain consistency with the framework developed for probability samples. The following theorem establishes identification assumptions for $\tau_{g}$ under $\beta_{0}$ and $\theta_{0}$.

Theorem 6 *Under conditions* **C2** *:* g(X) *only relies on* $X_{𝒞}$ *;* **C3** *:* τ(X)=E(Y|X,T=1,R=1)−E(Y|X,T=0,R=1) *; and* **C4** *:* $R⊥\tau \left(X\right)|X_{𝒞}$, *we have*

$$\tau_{g}=\frac{E\left[T_{i}\omega_{i}\left(\beta_{0},\theta_{0}\right)Y_{i}\right]}{E\left[T_{i}\omega_{i}\left(\beta_{0},\theta_{0}\right)\right]}-\frac{E\left[\left(1-T_{i}\right)\omega_{i}\left(\beta_{0},\theta_{0}\right)Y_{i}\right]}{E\left[\left(1-T_{i}\right)\omega_{i}\left(\beta_{0},\theta_{0}\right)\right]}.$$

In Theorem 6, Condition **C2** requires the target sub‐population be defined solely based on the covariates observed in the reference dataset. For estimating the PSATE, **C2** is verifiable because $g\left(X\right)=1_{ℬ}\left(X\right)$ is specified by the researchers. When estimating the PSATT and PSATO, assessing this condition is difficult because g(x) is a function of the unknown PS. Indeed, in the non‐probability setting with partially observed covariates, the target populations for the PSATT and PSATO cannot be clearly defined using the reference data. This suggests that we should focus on the estimation of the PSATE. Condition **C3** is weaker than the usual ignorability assumption for R with respect to Y given T, the one mentioned at the beginning of this section. Condition **C4** implies that it is not necessary to observe all the confounders in ${𝒮}_{B}$, but rather those associated with the CATE and the selection mechanism. Plausibility of **C3** relies on our knowledge of the CATE function. Under **C3**, the contribution of confounding variables to the CATE can be preliminarily identified by examining the coefficients of treatment‐by‐covariate interaction terms in a linear regression model on Y given X, T and their interactions. An example is given in Section 6.

For post‐treatment data, it is possible to observe the treatment indicator T in ${𝒮}_{B}$. When the confounders X are fully observed in ${𝒮}_{B}$, **C2** and **C4** must hold and by including T in the participation probability model, **C1** can be removed for identification. However, when the confounders are only partially observed in ${𝒮}_{B}$, including T in the participation probability model will ruin the identifiability provided by Theorem 6. That is, under **C2** and **C3**, the SW‐IPW estimator under $\beta_{0}$ and $\theta_{0}$ are biased unless $X_{𝒞}=X$. Therefore, we do not suggest including T for the estimation of the participation probabilities even if the treatment indicator is observed in ${𝒮}_{B}$ unless X are fully observed in ${𝒮}_{B}$.

Based on the above results, **C1**–**C4** are sufficient for identification of $\tau_{g}$ given a reference probability sample with partially observed confounding variables. It turns out that **C1** is not required for identifying the PSATE, as one can instead estimate the local PS when g(X) does not depend on the PS. Further details are provided in Section 4.5.

In practice, auxiliary information of the variables contributing significantly to treatment effect heterogeneity may be scattered into two or multiple reference datasets. This motivates us to consider an important research problem of combining multiple existing probability samples for more reliable estimation of the participation probabilities.

### 4.3 Estimation of Participation Probabilities Using Multiple Reference Samples

Suppose that we have J independent reference probability samples ${𝒮}_{B_{j}},j=1,\cdots ,J$, each of them may include a different set of covariates. Let ${𝒞}_{j}$ be the set of indices for the covariates observed in ${𝒮}_{B_{j}}$ with cardinality $p_{j}=\;|{𝒞}_{j}|$. We use $q_{j}$ to denote the sampling design for ${𝒮}_{B_{j}}$. Let $𝒞=\cup_{j=1}^{J}{𝒞}_{j}$ be the combined set of fully observed covariates among all J reference samples with set size $p_{c}=\;|𝒞|$. Without loss of generality, we assume that $𝒞=\left\{1,\cdots ,p_{c}\right\}$. For the j‐th reference sample, we observe the data $\left\{X_{{𝒞}_{j},i},d_{i}^{B_{j}},i\in {𝒮}_{B_{j}}\right\}$ where $d_{i}^{B_{j}},i\in {𝒮}_{B_{j}}$ are known survey weights for ${𝒮}_{B_{j}}$. We also assume that 1 is the first component of $X_{{𝒞}_{j}}$.

We consider a generalized linear model for the participation probabilities as $\pi_{\theta }\left(X_{𝒞}\right)=f\left(\theta^{⊤}X_{𝒞}\right)$ where f(⋅) is an inverse link function. Note that the combined set of covariates $X_{𝒞}$ is available in the non‐probability sample ${𝒮}_{A}$ but is not fully observed in any of the single reference sample ${𝒮}_{B_{j}}$. The likelihood function of θ is given by

$$\ell_{N}\left(\theta \right)=\sum_{i\in {𝒮}_{A}}\log\left\{\frac{\pi_{\theta }\left(X_{𝒞,i}\right)}{1-\pi_{\theta }\left(X_{𝒞,i}\right)}\right\}+\sum_{i=1}^{N}\log\left\{1-\pi_{\theta }\left(X_{𝒞,i}\right)\right\}.$$

Unfortunately, the pseudo‐maximum‐likelihood approach of Chen et al. [21] is no longer feasible because the second term of $\ell_{N}\left(\theta \right)$ cannot be approximated using the J reference samples. To circumvent this difficulty, we propose to use the following set of unbiased estimating equations for θ,

(9) $$0=\sum_{i=1}^{N}\left\{\frac{R_{i}}{\pi_{\theta }\left(X_{𝒞,i}\right)}-1\right\}X_{𝒞,i},$$

which reduces to the conventional calibration equations in survey sampling in the form of $\sum_{i\in {𝒮}_{A}}X_{𝒞,i}/\pi_{\theta }\left(X_{𝒞,i}\right)=\sum_{i=1}^{N}X_{𝒞,i}$ If we had access to the population totals $T_{𝒞}=\sum_{i=1}^{N}X_{𝒞,i}$ from census data, an estimate of θ can be obtained by solving (9). Under the current setting, each component of $T_{𝒞}={\left(T_{1},\cdots ,T_{p_{c}}\right)}^{⊤}$ can be estimated from at least one of the reference samples. More generally, for each k∈𝒞, let ${\hat{T}}_{\mathrm{kj}}=\sum_{i\in {𝒮}_{B_{j}}}d_{i}^{B_{j}}X_{k,i}$ be the estimate for $T_{k}$ if $X_{k,i}$ is available in ${𝒮}_{B_{j}}$. Then, a weighted average of ${\hat{T}}_{\mathrm{kj}}$ for $j:k\in {𝒞}_{j}$ would be an unbiased and consistent estimator of the true population total $T_{k}$. The proposed estimator $\hat{\theta }$ of θ is obtained by solving

(10) $$0=\sum_{i\in {𝒮}_{A}}\frac{X_{k,i}}{\pi_{\theta }\left(X_{𝒞,i}\right)}-\sum_{j:k\in {𝒞}_{j}}a_{\mathrm{kj}}{\hat{T}}_{\mathrm{kj}},\;k=1,\cdots ,p_{c},$$

where $a_{\mathrm{kj}}\geq 0$ for $k\in {𝒞}_{j}$, j=1,⋯,J satisfy $\sum_{j:k\in {𝒞}_{j}}a_{\mathrm{kj}}=1$. An optimal choice for $a_{\mathrm{kj}}$ in terms of estimating $T_{k}$ using available data is to make it proportional to the inverse of the estimated design‐based variance of ${\hat{T}}_{\mathrm{kj}}$. Another simple choice in practice is $a_{\mathrm{kj}}=n_{B_{j}}/\sum_{i:k\in {𝒞}_{i}}n_{B_{i}}$ where $n_{B_{j}}$ is the sample size of ${𝒮}_{B_{j}}$.

### 4.4 Variance Estimation

Consistent estimators of $\beta_{0}$ and $\theta_{0}$ lead to a consistent estimator of $\tau_{g}$. Even when the models for the PS and the participation probability are mis‐specified, the selection and confounding bias in causal inference are expected to be reduced by survey‐weighted propensity score weighting. However, the estimation of θ yields inflated variance of the estimator for $\tau_{g}$. In this section, we provide a variance estimator for $\tau_{g}$ under the setting that the population size N is much bigger than n and $n_{B_{j}},j=1,\cdots ,J$.

Let $A_{j}={\left\{a_{\mathrm{kj}}\right\}}_{k=1}^{p_{c}}$ be the $p_{c}\times p_{c}$ diagonal matrix, j=1,⋯,J where $a_{\mathrm{kj}}$ were defined in Section 4.3. We let the unobserved covariates in a particular reference sample be zero, that is, $X_{k,j}=0$ for $k\notin {𝒞}_{j}$. Let $\eta ={\left(\theta^{⊤},\beta^{⊤},\tau_{1},\tau \right)}^{⊤}$. It follows that the proposed survey‐weighted estimation procedure with non‐probability samples can be formulated solving the following system of equations:

(11) $$0=S_{n}\left(\eta \right)=\frac{1}{N}\sum_{i\in {𝒮}_{A}}\frac{\psi_{A}\left(O_{i},\eta \right)}{\pi_{\theta }\left(X_{𝒞,i}\right)}-\frac{1}{N}\sum_{j=1}^{J}\sum_{i\in {𝒮}_{B_{j}}}d_{i}^{B_{j}}\psi_{B_{j}}\left(X_{𝒞,i}\right),$$

where

$$\psi_{A}\left(O_{i},\eta \right)=\left\{\begin{array}{c} X_{i}\\ h\left(X_{i},T_{i},\beta \right)\\ \frac{T_{i}g\left(X_{i}\right)}{e_{\beta }\left(X_{i}\right)}\left(Y_{i}-\tau_{1}\right)\\ \frac{\left(1-T_{i}\right)g\left(X_{i}\right)}{1-e_{\beta }\left(X_{i}\right)}\left(Y_{i}-\tau_{1}+\tau \right) \end{array}\right\}$$

and $\psi_{B_{j}}\left(X_{𝒞,i}\right)={\left(X_{𝒞,i}^{⊤}A_{j},0,0,0\right)}^{⊤}$. We assume that the sizes of the J probability samples, $n_{B_{j}}$, are of the same order as n. Let $\eta^{*}$ denote the probability limit of $\hat{\eta }$ under the joint model. Under suitable regularity conditions, we have $\hat{\eta }-\eta^{*}=E{\left[\partial S_{n}\left(\eta^{*}\right)/\partial \eta^{⊤}\right]}^{-1}S_{n}\left(\eta^{*}\right)+o_{p}\left(n^{-1/2}\right)$. It follows that $V\left(\hat{\eta }\right)=D^{-1}V\left\{S_{n}\left(\eta^{*}\right)\right\}D^{-1}+o_{p}\left(n^{-1}\right)$ where $D=E\left[\partial S_{n}\left(\eta^{*}\right)/\partial \eta^{⊤}\right]$. A variance estimator of $\hat{\eta }$ is obtained by plugging in an estimator for D and an estimator for $V\left\{S_{n}\left(\eta^{*}\right)\right\}$ in the sandwich form. The first term can be consistently estimated by ${\hat{N}}^{-1}\sum_{i\in {𝒮}_{A}}{\left({\hat{\pi }}_{i}^{A}\right)}^{-1}\partial \psi_{A}\left(O_{i},\hat{\eta }\right)/\partial \eta^{⊤}$ where ${\hat{\pi }}_{i}^{A}=\pi_{\hat{\theta }}\left(X_{𝒞,i}\right)$ and $\hat{N}=\sum_{i\in {𝒮}_{A}}{\left({\hat{\pi }}_{i}^{A}\right)}^{-1}=\sum_{j=1}^{J}a_{1j}\sum_{i\in {𝒮}_{B_{j}}}d_{i}^{B_{j}}$. For the second term, by noting that

$$\frac{1}{N^{2}}V\left\{E_{q_{j}}\left[\sum_{i\in {𝒮}_{B_{j}}}d_{i}^{B_{j}}\psi_{B_{j}}\left(X_{𝒞,i}\right)\right]\right\}=\frac{1}{N^{2}}V\left\{\sum_{i=1}^{N}\psi_{B_{j}}\left(X_{𝒞,i}\right)\right\}=O\left(N^{-1}\right)=o\left(n^{-1}\right)$$

for j=1,⋯,J under suitable regularity conditions, we have

$$V\left\{S_{n}\left(\eta^{*}\right)\right\}=\frac{1}{N}V\left\{\frac{R\psi_{A}\left(O,\eta^{*}\right)}{\pi_{\theta^{*}}\left(X_{𝒞}\right)}\right\}+\frac{1}{N^{2}}\sum_{j=1}^{J}E\left[V_{q_{j}}\left\{\sum_{i\in {𝒮}_{B_{j}}}\psi_{B_{j}}\left(X_{𝒞,i}\right)\right\}\right]+o_{p}\left(n^{-1}\right),$$

which can be estimated by

$$\begin{array}{cc} & \frac{1}{{\hat{N}}^{2}}\sum_{i\in {𝒮}_{A}}\frac{\psi_{A}{\left(O_{i},\hat{\eta }\right)}^{⊗2}}{{\left({\hat{\pi }}_{i}^{A}\right)}^{2}}-\frac{1}{{\hat{N}}^{3}}{\left\{\sum_{i\in {𝒮}_{A}}\frac{\psi_{A}\left(O_{i},\hat{\eta }\right)}{{\hat{\pi }}_{i}^{A}}\right\}}^{⊗2}\\ & \;+\frac{1}{{\hat{N}}^{2}}\sum_{j=1}^{J}\sum_{i\in {𝒮}_{B_{j}}}\;\sum_{k\in {𝒮}_{B_{j}}}\frac{\pi_{\mathrm{ik}}^{B_{j}}-\pi_{i}^{B_{j}}\pi_{k}^{B_{j}}}{\pi_{\mathrm{ik}}^{B_{j}}\pi_{i}^{B_{j}}\pi_{k}^{B_{j}}}\psi_{B_{j}}{\left(X_{𝒞,i}\right)}^{⊗2}. \end{array}$$

Similar to discussions in Section 3.3, approximate variance estimators without involving second order inclusion probabilities can be used in practice, and the validity of the variance estimator does not require correct specification of the PS and the participation probability models.

### 4.5 Handling Pre‐Treatment Non‐Probability Samples

Our discussion so far has focused on post‐treatment observational data. However, pre‐treatment data are also common in observational studies, particularly in the context of non‐probability samples, where study participants may be self‐recruited or selected based on volunteering or expert judgment prior to treatment assignment. In this subsection, we demonstrate that the proposed method for post‐treatment data is also applicable to pre‐treatment non‐probability samples.

An important feature of pre‐treatment observational data is that the treatment indicators are not conceptually well‐defined for samples excluded, nor are the PS function and the population causal estimands defined with respect to the PS such as the PSATT and PSATO. Accordingly, we assume that $\left\{\left(Y_{i}\left(1\right),Y_{i}\left(0\right),X_{i},R_{i}\right),i\in {𝒰}_{N}\right\}$ are N i.i.d. copies of (Y(1),Y(0),X,R), and that $\left\{T_{i},i\in {𝒮}_{A}\right\}$ are i.i.d. draws from the conditional distribution of T∣R=1. We assume local ignorability: {Y(1),Y(0)}⊥T∣X,R=1 and define the local PS as $e_{l}\left(x\right)=P\left(T=1|X=x,R=1\right)$. We further assume that $0<e_{l}\left(x\right)<1$ for x∈𝒳 and focus on the estimation of the PSATE where $g\left(X\right)=1_{ℬ}\left(X\right)$.

The methodology part remains the same. The only difference lies in that the parametric model $e_{\beta }\left(x\right)$ is postulated for the local PS $e_{l}\left(x\right)$. Therefore, we need to readdress identification assumptions for $\beta_{0}$ and $\tau_{g}$.

Theorem 7 *When* $h=h_{\mathrm{BL}}$ *or* $h=h_{\mathrm{CB},g}$, *we have* $E\left\{\left[R/\pi_{\theta_{0}}\left(X_{𝒞}\right)\right]h\left(X,T,\beta_{0}\right)\right\}=0$ *and* $E\left[Rh\left(X,T,\beta_{0}\right)\right]=0$.

Theorem 7 follows directly from the fact that $E\left[h\left(X,T,\beta_{0}\right)|X,R=1\right]=0$ when g(X) does not involve the PS. In other words, we do not require **C1** to identify the local PS.

Theorem 8 *Under* **C2** *and* **C4** *given in Theorem* 6 *and* **E3** *:* τ(X)=E[Y(1)−Y(0)∣X,R=1], *we have*

$$\tau_{g}=\frac{E\left[T_{i}\omega_{i}\left(\beta_{0},\theta_{0}\right)Y_{i}\right]}{E\left[T_{i}\omega_{i}\left(\beta_{0},\theta_{0}\right)\right]}-\frac{E\left[\left(1-T_{i}\right)\omega_{i}\left(\beta_{0},\theta_{0}\right)Y_{i}\right]}{E\left[\left(1-T_{i}\right)\omega_{i}\left(\beta_{0},\theta_{0}\right)\right]}.$$

*Conditions **E3** and **C4** can be replaced by **E4**:* $R⊥\left\{Y\left(1\right)-Y\left(0\right)\right\}|X_{𝒞}$, *and the proposition still holds*.

Condition **E4** is slightly stronger than **C4**, with the additional requirement given by **E3**. We replace **C3** by **E3** in the pre‐treatment setting because in this subsection we assumed local ignorability for the potential outcomes. The proof is given in the Supporting Information. Theorems 7 and 8 justify the application of the proposed approach to pre‐treatment observational data.

## 5 Numerical Studies

We design and conduct simulation studies under settings for probability survey samples and non‐probability samples, in the context of observational studies, to evaluate the finite‐sample performance of proposed methods. For probability survey samples, we focus on the estimation accuracy and the coverage probabilities of the associated inferential procedures when the postulated models are slightly misspecified. For non‐probability samples, we focus on identification under partial observations of confounding variables in the reference datasets.

### 5.1 Simulations With Probability Survey Samples

We first conduct simulations under probability sampling designs. The setup of the simulations is as follows. We generate a group of covariates $X_{i}={\left(X_{1i},X_{2i},X_{3i},X_{4i},X_{5i}\right)}^{⊤}$ by $X_{1i}=Z_{1i}$, $X_{2i}=Z_{2i}+0.3X_{1i}$, $X_{3i}=Z_{3i}+0.2\left(X_{1i}+X_{2i}\right)$, $X_{4i}=Z_{4i}+0.1\left(X_{1i}+X_{2i}+X_{3i}\right)$, $X_{5i}=Z_{5i}^{2}+0.2X_{3i}X_{4i}$ where $Z_{1i}∼\text{Bernoulli}\left(0.5\right)$, $Z_{2i}∼\text{Unif}\left(0,4\right)$, $Z_{3i}∼\mathrm{Exp}\left(1\right)$, $Z_{4i}∼\chi \left(4\right)$, and $Z_{5i}∼N\left(0,2^{2}\right)$ so that the covariates are all positive (the auxiliary variables of a survey are typically positive) and mildly correlated with each other. We generate the treatment indicators $T_{i}$ from the binomial distribution with the PS defined as

$$e\left(X_{i}\right)=\text{expit}\left(-X_{1i}+0.6X_{2i}-0.5X_{3i}+0.1X_{4i}+\beta_{0}\right),$$

where $\beta_{0}$ is chosen so that $\sum_{i=1}^{N}e\left(X_{i}\right)=1/2$. The observed outcomes are generated from

$$Y_{i}=m_{0}\left(X_{i}\right)+T_{i}\tau \left(X_{i}\right)+N\left(0,2^{2}\right)$$

where $m_{0}\left(X_{i}\right)=X_{1i}+X_{2i}+X_{4i}+X_{5i}+2$, $\tau \left(X_{i}\right)=0.5m\left(X_{i}\right)+\beta_{\tau }$, and $\beta_{\tau }$ is chosen so that the PATE is equal to 3. Now, we have created the finite‐population data ${\left\{\left(Y_{i},T_{i},X_{i}\right)\right\}}_{i=1}^{N}$. We set N=2e4. The survey sample ${𝒮}_{A}$ is selected from the finite population by the randomized systematic PPS sampling method with the sample size n=500 and the first inclusion probability $\pi_{i}^{A}$ set to be proportional to $X_{4i}+c$ where c is a controlling factor so that $\max_{i}\left\{X_{4i}+c\right\}/\min_{i}\left\{X_{4i}+c\right\}=50$ [45]. We replicate this procedure for B=1000 times and the simulation results are based on such 1000 survey samples. The outcome $Y_{i}$ and the treatment $T_{i}$ are always observed for ${𝒮}_{A}$ while the covariates $X_{i}$ may not. Four different scenarios are studied:

- **TT**: $X_{1i},X_{2i},X_{3i},X_{4i},X_{5i}$ are fully observed.
- **TF**: $X_{1i},X_{2i},X_{4i},X_{5i}$ are observed.
- **FT**: $X_{1i},X_{2i},X_{3i},X_{4i}$ are observed.
- **FF**: $X_{1i},X_{2i},X_{4i}$ are observed.

We say the outcome model is correct if $m_{0}\left(X_{i}\right)$ is linear in the observed covariates. **TT** yields correct outcome and PS models; **TF** yields correct outcome model but wrong PS model; **FT** yields correct PS model but wrong outcome model; **FF** yields two wrong models.

We focus on the SW‐IPW estimators obtained as the solution for τ in Equation (5), with weights constructed using different approaches. When the survey weights in (5) are set to one, the SW‐IPW estimators reduce to the usual IPW estimators and β is estimated based on either maximum likelihood estimation (MLE) or CBPS. When the survey weights are incorporated, β is estimated based on either MPLE or SW‐CBPS. For comparison, we also include the difference‐in‐means estimator as a benchmark, referred to as “Naive.” We estimate the PATE, PATT, and PSATE with the sub‐population $ℬ=\left\{2<X_{4i}<7\right\}$. To evaluate these methods, we consider the following performance measures: (1) %RB: percentage relative bias for the point estimation, that is, $\%\mathrm{RB}=1/B\sum_{b=1}^{B}\left({\hat{\tau }}^{\left(b\right)}-\tau_{g}\right)/\tau_{g}\times 100$; (2) MCSD: Monte Carlo standard deviation; (3) AVESE: average of estimated standard errors; (4) RMSE: root mean squared error; (5) $\%\mathrm{CP}=1/B\sum_{b=1}^{B}1\left\{{\hat{\tau }}^{\left(b\right)}\in C_{0.25}\right\}$: percentage empirical coverage probability for the 95% confidence interval. The simulation results for estimating the PATE, the PATT, and the PSATE are presented in Tables 2, 3, 4, respectively. The findings based on these tables are summarized as follows.

**TABLE 2:** Simulation results for estimating the PATE with probability survey samples.

Scenario	Method	%RB	MCSD	AVESE	RMSE	%CP
TT	Naive	89.96	0.86	0.88	2.83	12.30
	MLE	41.33	0.44	0.43	1.31	14.40
	CBPS	40.19	0.30	0.28	1.24	0.60
	MPLE	2.02	0.48	0.47	0.48	95.40
	SW‐CBPS	2.42	0.32	0.30	0.33	92.40
TF	Naive	89.96	0.86	0.88	2.83	12.30
	MLE	44.11	0.39	0.40	1.38	4.30
	CBPS	40.76	0.28	0.27	1.25	0.20
	MPLE	3.25	0.42	0.43	0.43	95.40
	SW‐CBPS	2.51	0.30	0.29	0.31	92.80
FT	Naive	89.96	0.86	0.88	2.83	12.30
	MLE	41.68	0.78	0.79	1.48	64.40
	CBPS	40.84	0.75	0.75	1.44	64.20
	MPLE	0.14	0.92	0.90	0.92	94.40
	SW‐CBPS	0.43	0.90	0.86	0.90	93.80
FF	Naive	89.96	0.86	0.88	2.83	12.30
	MLE	17.12	0.74	0.75	0.90	90.80
	CBPS	14.54	0.73	0.74	0.85	92.00
	MPLE	−18.84	0.85	0.85	1.02	85.90
	SW‐CBPS	−19.34	0.85	0.84	1.03	85.10

**TABLE 3:** Simulation results for estimating the PATT with probability survey samples.

Scenario	Method	%RB	MCSD	AVESE	RMSE	%CP
TT	Naive	77.28	0.86	0.88	2.63	17.80
	MLE	45.21	0.64	0.62	1.59	27.50
	CBPS	41.02	0.33	0.33	1.36	1.70
	MPLE	−0.38	0.54	0.56	0.54	95.70
	SW‐CBPS	1.90	0.35	0.34	0.35	93.80
TF	Naive	77.28	0.86	0.88	2.63	17.80
	MLE	41.03	0.51	0.52	1.41	25.50
	CBPS	40.28	0.32	0.32	1.33	1.50
	MPLE	−3.17	0.46	0.47	0.47	95.50
	SW‐CBPS	1.44	0.34	0.34	0.34	94.20
FT	Naive	77.28	0.86	0.88	2.63	17.80
	MLE	45.03	0.88	0.88	1.69	57.20
	CBPS	42.90	0.79	0.78	1.59	56.10
	MPLE	−1.68	0.86	0.87	0.86	94.70
	SW‐CBPS	1.02	0.84	0.83	0.84	94.60
FF	Naive	77.28	0.86	0.88	2.63	17.80
	MLE	22.51	0.83	0.84	1.10	84.90
	CBPS	18.62	0.78	0.77	0.98	88.60
	MPLE	−18.02	0.83	0.85	1.01	89.00
	SW‐CBPS	−16.42	0.81	0.81	0.97	89.40

**TABLE 4:** Simulation results for estimating the PSATE with probability survey samples.

Scenario	Method	%RB	MCSD	AVESE	RMSE	%CP
TT	Naive	96.83	0.86	0.88	3.03	7.80
	MLE	8.46	0.80	0.76	0.84	93.00
	CBPS	8.65	0.34	0.32	0.43	85.60
	MPLE	−0.21	0.84	0.81	0.84	94.30
	SW‐CBPS	−0.12	0.35	0.33	0.35	93.80
TF	Naive	96.83	0.86	0.88	3.03	7.80
	MLE	16.29	0.82	0.78	0.95	90.70
	CBPS	9.53	0.32	0.31	0.43	84.70
	MPLE	3.07	0.83	0.81	0.84	94.80
	SW‐CBPS	0.47	0.33	0.32	0.33	94.30
FT	Naive	96.83	0.86	0.88	3.03	7.80
	MLE	9.12	1.02	0.99	1.05	94.20
	CBPS	7.92	0.96	0.92	0.99	94.30
	MPLE	−2.27	1.09	1.05	1.10	94.80
	SW‐CBPS	−2.11	1.02	0.97	1.03	94.40
FF	Naive	96.83	0.86	0.88	3.03	7.80
	MLE	−9.13	0.98	0.96	1.01	93.10
	CBPS	−10.54	0.93	0.91	0.98	92.20
	MPLE	−18.88	1.04	1.02	1.19	89.80
	SW‐CBPS	−18.88	0.99	0.96	1.14	87.20

First, the Naive, MLE, and CBPS methods are biased for all three estimands. The biases are substantially reduced after incorporating the survey weights as expected. In the scenario where the PS model is wrongly specified (**TF**), MPLE presents slight bias (around 3%), and the relative bias of SW‐CBPS is less than 1.5% for the PATT and PSATE. This reveals that SW‐CBPS is robust against model mis‐specification as described in Theorem 3. When at least one model is correctly specified, SW‐CBPS outperforms other methods in terms of Bias and RMSE. For estimating the PATE, the variances of the SW‐CBPS estimators are underestimated, and the coverage rates fall below the nominal level but remain within an acceptable range.

### 5.2 Simulations With Non‐Probability Survey Samples

We slightly modify the setup of the simulations with probability survey samples to evaluate the performance of the proposed methods with non‐probability samples. We redo the data generating process given in Section 5.1 for the finite population data excepting that $\tau \left(X_{i}\right)$ is reset and will be identified later. The survey sample ${𝒮}_{A}$ is selected based on the Poisson sampling method with the first inclusion probability

$$\pi_{i}^{A}=\text{expit}\left(0.1X_{1i}+0.1X_{2i}+0.1X_{3i}+0.1X_{4i}+T_{i}+\beta_{A}\right)$$

where $\beta_{A}$ is a controlling parameter such that $\sum_{i=1}^{N}\pi_{i}^{A}=n$. We create three reference probability samples, ${𝒮}_{B_{1}}$, ${𝒮}_{B_{2}}$, and ${𝒮}_{B_{3}}$, with the sample sizes $n_{B_{1}}=n_{B_{2}}=n_{B_{3}}=1000$, based on the randomized systematic PPS sampling (without replacement) method with the first inclusion probabilities $\pi_{i}^{B_{1}}$, $\pi_{i}^{B_{2}}$, and $\pi^{B_{3}}$, respectively. Both $\pi_{i}^{B_{1}}$ and $\pi_{i}^{B_{3}}$ are set to be proportional to $X_{3i}+c$ where c is chosen so that $\max_{i}\left\{X_{3i}+c\right\}/\min_{i}\left\{X_{3i}+c\right\}=50$. The $\pi_{i}^{B_{2}}$ is proportional to $X_{4i}+c$ where c is set to satisfy $\max_{i}\left\{X_{4i}+c\right\}/\min_{i}\left\{X_{4i}+c\right\}=50$. However, the observed auxiliary variables are different for the three reference samples. We consider two different designs as summarized in Table 5. The population and sample size are (N,n)=(2e4,500).

**TABLE 5:** Designs for simulations with non‐probability survey samples.

Design	Observed covariates			$\tau \left(X_{i}\right)$
	SB1	SB2	SB3	
I	$X_{1i},X_{2i}$	$X_{1i},X_{4i}$	Same as ${𝒮}_{A}$	$X_{1i}+X_{2i}+0.2X_{4i}+\beta_{\tau }$
II	$X_{2i}$	$X_{1i},X_{3i},X_{4i}$		$X_{2i}+\beta_{\tau }$

Similarly, we focus on the SW‐IPW estimator. The additional step is to estimate the participation probabilities $\pi_{i}^{A}=\pi \left(X_{𝒞,i}\right)$ using the calibration approach presented in Section 4.3 where external reference data are required. We consider four scenarios for the available external information.

- SB1: ${𝒮}_{B_{1}}$ is available.
- SB2: ${𝒮}_{B_{2}}$ is available.
- SB3: ${𝒮}_{B_{3}}$ is available.
- SB12: ${𝒮}_{B_{1}}$ and ${𝒮}_{B_{2}}$ are both available.

SB3 contains the information of all the confounding variables in ${𝒮}_{A}$. SB1 and SB2 represent the scenarios where the confounding variables are only partially observed. For comparison, we also include the estimators of $\tau_{g}$ obtained from solving (5) as if we know the inclusion probabilities, referred to as “Oracle,” and the case that we set the survey weights uniformly to one, referred to as “Unif.” The two designs as described in Table 5 are for different purposes of the evaluation. The main focus of Design I is to figure out whether the estimator of $\tau_{g}$ can be improved by borrowing information from multiple reference samples (SB12), while the objective of Design II is to study which covariates play the key role in identification. We evaluate these methods under the scenarios **TT**, **TF**, and **FT** where at least one model is correct. We focus on estimating the PATE and calculate the performance measures based on 1000 Monte Carlo replicated datasets. The simulation results for Design I and Design II are summarized respectively in Tables 6 and 7.

**TABLE 6:** Simulation results with non‐probability survey samples under Design I.

Scenario	Method	MPLE					SW‐CBPS				
		%RB	MCSD	AVESE	RMSE	%CP	%RB	MCSD	AVESE	RMSE	%CP
TT	Naive	61.27	0.83	0.83	2.01	41.30	61.27	0.83	0.83	2.01	41.30
	Unif	23.06	0.67	0.61	0.96	59.00	20.86	0.28	0.25	0.68	33.60
	Oracle	1.94	0.39	0.40	0.39	94.60	2.39	0.26	0.25	0.27	93.60
	SB1	12.95	0.61	0.59	0.72	76.40	11.36	0.27	0.24	0.43	67.50
	SB2	9.77	0.49	0.50	0.57	83.40	11.58	0.27	0.25	0.44	68.80
	SB3	0.45	0.54	0.49	0.54	93.60	2.42	0.27	0.24	0.28	92.20
	SB12	0.33	0.46	0.48	0.46	94.20	2.41	0.26	0.24	0.27	92.60
TF	Naive	61.27	0.83	0.83	2.01	41.30	61.27	0.83	0.83	2.01	41.30
	Unif	19.98	0.48	0.46	0.76	63.00	20.27	0.26	0.24	0.66	31.60
	Oracle	2.26	0.33	0.35	0.34	95.30	1.63	0.25	0.24	0.25	93.90
	SB1	9.95	0.44	0.44	0.53	81.70	10.56	0.25	0.23	0.40	70.50
	SB2	7.06	0.39	0.39	0.45	86.90	11.08	0.25	0.24	0.42	69.20
	SB3	−2.10	0.44	0.39	0.45	95.60	1.67	0.25	0.23	0.26	93.30
	SB12	−2.33	0.37	0.38	0.38	95.90	1.69	0.25	0.23	0.25	93.20
FT	Naive	61.27	0.83	0.83	2.01	41.30	61.27	0.83	0.83	2.01	41.30
	Unif	21.25	0.90	0.84	1.10	79.70	20.39	0.75	0.71	0.97	79.30
	Oracle	0.39	0.74	0.74	0.74	94.70	1.07	0.71	0.70	0.71	93.90
	SB1	11.90	0.84	0.81	0.91	88.40	11.27	0.72	0.70	0.79	88.80
	SB2	7.64	0.78	0.77	0.81	91.80	9.94	0.73	0.71	0.79	89.40
	SB3	−0.70	0.79	0.75	0.79	94.70	1.34	0.72	0.70	0.72	94.40
	SB12	−1.03	0.75	0.75	0.75	94.70	1.22	0.71	0.70	0.71	94.10

**TABLE 7:** Simulation results with non‐probability survey samples under Design II.

Scenario	Method	MPLE					SW‐CBPS				
		%RB	MCSD	AVESE	RMSE	%CP	%RB	MCSD	AVESE	RMSE	%CP
TT	Naive	53.37	0.82	0.82	1.79	49.40	53.37	0.82	0.82	1.79	49.40
	Unif	15.17	0.67	0.61	0.81	72.40	13.08	0.27	0.25	0.48	59.90
	Oracle	1.73	0.38	0.40	0.39	94.20	2.20	0.26	0.24	0.27	93.20
	SB1	5.02	0.61	0.59	0.63	87.30	3.44	0.26	0.24	0.28	90.80
	SB2	9.17	0.50	0.51	0.57	84.80	10.85	0.27	0.25	0.42	71.20
	SB3	0.36	0.54	0.49	0.54	93.80	2.27	0.26	0.24	0.27	92.50
	SB12	0.27	0.46	0.49	0.46	94.20	2.30	0.26	0.24	0.27	93.30
TF	Naive	53.37	0.82	0.82	1.79	49.40	53.37	0.82	0.82	1.79	49.40
	Unif	12.09	0.48	0.46	0.60	78.30	12.52	0.26	0.24	0.45	61.20
	Oracle	2.12	0.33	0.34	0.33	95.60	1.52	0.25	0.24	0.25	93.80
	SB1	2.05	0.45	0.44	0.45	92.10	2.69	0.25	0.23	0.26	92.20
	SB2	6.96	0.39	0.39	0.44	87.00	10.96	0.25	0.24	0.41	68.90
	SB3	−2.18	0.42	0.39	0.42	96.00	1.58	0.25	0.23	0.25	92.60
	SB12	−2.24	0.39	0.38	0.40	95.70	1.60	0.24	0.24	0.25	93.90
FT	Naive	53.37	0.82	0.82	1.79	49.40	53.37	0.82	0.82	1.79	49.40
	Unif	13.36	0.90	0.84	0.98	86.10	12.60	0.75	0.71	0.84	86.30
	Oracle	0.21	0.74	0.74	0.74	94.70	0.90	0.71	0.70	0.71	94.00
	SB1	3.94	0.84	0.82	0.85	92.20	3.38	0.72	0.70	0.72	92.20
	SB2	7.28	0.79	0.77	0.82	91.70	9.40	0.74	0.71	0.79	89.60
	SB3	−0.88	0.77	0.75	0.77	94.40	1.12	0.71	0.70	0.71	94.10
	SB12	−1.08	0.76	0.76	0.76	94.50	1.08	0.71	0.70	0.71	94.50

From Table 6, it can be seen that the Unif estimators are biased. The biases are mildly corrected after involving either ${𝒮}_{B_{1}}$ or ${𝒮}_{B_{2}}$ for correction and substantially removed after integrating ${𝒮}_{B_{1}}$ and ${𝒮}_{B_{2}}$ together (SB12). SB12 has similar performance as SB3 in terms of Bias, and outperforms SB3 in terms of RMSE. Especially, when the PS function is estimated by the CBPS method, SB12 is as good as Oracle. This numerical experiment showcases the advantages of integrating multiple reference datasets for the estimation when a single reference sample cannot provide the required auxiliary information: In our setup, SB12 outperforms SB1, SB2 and SB3 in terms of both Bias and RMSE.

In Design II, the participation probabilities are expected to be better estimated using ${𝒮}_{B_{2}}$ than ${𝒮}_{B_{1}}$ because ${𝒮}_{B_{2}}$ contains more important predictors in the true participation probabilities. However, remember that our ultimate goal is to estimate the population average treatment effects rather than the participation probabilities. As argued in Theorem 6, ${𝒮}_{B_{1}}$ is preferred and also sufficient for identification of $\tau_{g}$ because the CATE function merely relies on $X_{2i}$. If we do not observe key variables for the CATE, we may combine ${𝒮}_{B_{1}}$ and ${𝒮}_{B_{2}}$ together to catch important predictors for the participation probabilities. Table 7 shows that SB1 removes more biases than SB2 as expected in Scenarios **TT**, **TF** and **FT**. However, since $\pi_{\theta }$ is wrongly specified by simply involving $X_{2i}$, SB1 is mildly biased. SB12 still achieves the best performance among all other methods in terms of Bias, RMSE, and coverage rate.

## 6 Real Data Analysis: Right Heart Catheterization

Hirano and Imben [2] analyzed the observational data from the Study to Understand Prognoses and Preferences for Outcomes and Risks of Treatments (SUPPORT). They combined propensity score weighting with linear regression to analyze the causal effect of Right Heart Catheterization (RHC) on survival. The sample contains 5735 heavily ill adults collected from 5 medical centers in the United States, among which 2184 individuals in the treated group received RHC during the first 24 hours of admission, and the remaining 3551 units are in the control group. A set of 72 confounding variables including some demographic factors and health condition measurements was recorded during the study based on a panel of experts. We further categorize the continuous variable “age” into four groups: age_lt30=1{18≤age<30}; age_30_49=1{30≤age<50}; age_50_69=1{50≤age<70}; age_ge70=1{age≥70}, leading to 75 confounding variables. The outcome is the indicator of survival at 30 days. After adjusting for the confounders, the previous studies concluded that RHC has a remarkably negative treatment effect on survival. However, they focused on the sample alone, which is apparently a pre‐treatment non‐probability sample.

In this section, we would like to address the research question: Can the causal conclusion be generalized to the target finite population? Based on our discussion in Section 4.5, the answer to this question depends on treatment effect heterogeneity of RHC across the confounding variables. If the CATE does not vary with the values of confounders, then the average treatment effect estimate is broadly applicable and does not rely on the population of interest. Otherwise, we need to further adjust for the variables contributing significantly to the CATE using the tool developed in Section 4. We assume that $m_{T}\left(X\right)=E\left[Y|R=1,T,X\right]$ and fit the following linear regression model with treatment‐by‐covariate interaction terms:

$$m_{T}\left(X\right)=\tau T+\alpha_{x}^{⊤}\left(X-{\bar{x}}_{n}\right)+\alpha^{⊤}T\left(X-{\bar{x}}_{n}\right),$$

where ${\bar{x}}_{n}$ is the vector of sample means of the covariates. Under this model, we have $\tau \left(X\right)=m_{1}\left(X\right)-m_{0}\left(X\right)=\tau +\alpha^{⊤}\left(X-{\bar{x}}_{n}\right)$. Thus, the effect of a confounding variable on the CATE can be assessed through the coefficient of the corresponding interaction with the treatment indicator. We implemented least squares regression in R through the function lm, where a part of the summary table of regression analysis is presented in Table 8. We find that among all the treatment‐by‐covariate interaction terms, only the coefficients of RHC:sex_Female and RHC:age_ge70 are statistically significant. This implies that we should focus on the marginal distributions of the variables sex_Female and age_ge70 when we extend the causal conclusion from the sample to the target population.

**TABLE 8:** Summary table of least squares regression.

	Estimate	SE	*z*	*p*
(Intercept)	0.37	0.01	47.50	0.000*
RHC	−0.06	0.01	−4.33	0.000*
edu	0.00	0.00	−1.33	0.183
cardiohx	−0.04	0.02	−1.65	0.100
chfhx	−0.10	0.02	−4.08	0.000*
dementhx	−0.05	0.02	−2.20	0.028*
		…		
RHC:edu	0.00	0.00	0.77	0.441
RHC:cardiohx	0.01	0.04	0.28	0.781
RHC:chfhx	0.01	0.04	0.18	0.855
RHC:dementhx	0.00	0.04	0.09	0.926
		…		
RHC:sex_Female	−0.05	0.03	−2.12	0.034*
RHC:age_lt30	0.06	0.08	0.67	0.503
RHC:age_30_49	0.06	0.07	0.86	0.391
RHC:age_50_69	0.09	0.06	1.50	0.134
RHC:age_ge70	0.13	0.06	2.04	0.042*

* The level of significance is 0.05.

We use the survey dataset from the Current Population Survey (CPS) in 2015 as the reference probability sample, with the target population defined as Adults of the United States in 2015. The 2015 CPS dataset contains three common variables as the SUPPORT dataset: age, an indicator of race black, and gender. Similarly, we break up the variable “age” into four binary indicators. Thus, the two datasets now have 6 shared variables. Table 9 compares the survey‐weighted estimates of the population means of the shared covariates in the CPS dataset with the corresponding sample means in the SUPPORT data (scaled by $10^{2}$), which are visibly different.

**TABLE 9:** Marginal distributions of the common variables for the CPS and the SUPPORT data.

	CPS	SUPPORT
race_black	12.5	16.0
sex_Female	51.8	44.3
age_lt30	21.2	5.0
age_30_49	31.9	18.5
age_50_69	31.4	37.8
age_ge70	12.6	35.0

We apply the proposed survey‐weighted propensity score weighting approach to estimate the PATE of RHC. We first estimate the participation probabilities for the sample units based on the calibration method discussed in Section 4.3, and then construct the weights as in (7) to create the SW‐IPW estimator for the PATE. We postulated a logistic model for the participation probability and consider two different sets of variables for modeling. We use either all the shared covariates in Table 9, denoted by var_set1, or var_set2 = {sex_Female, age_ge70}. The weighted group means of some covariates under the methods MLE and MPLE are presented in Table 10, where we also include unweighted group means and the survey‐weighted means from the CPS dataset for comparison. It can be seen that the covariates are better balanced between the treated and control groups after weighting under MLE, while they are still imbalanced between the treatment groups and the target population. As we incorporate the survey weights and apply the MPLE method under var_set1 or var_set2, the weighted means of the variables in var_set1 or var_set2 have been adjusted closer to the survey‐weighted means and the confounding variables remain balanced between the treated and control groups. The estimates of the PATE based on different methods are given in Table 11. After incorporating the survey weights to correct for selection bias, the estimated average treatment effect becomes even more negative and the variance of the estimator gets inflated, indicating that the causal effect of RHC is also negative with respect to the target population. When the set of covariates used in the participation probability model is reduced from var_set1 to var_set2, the standard error of the estimate also decreases.

**TABLE 10:** Weighted means of the covariates in the treated and control groups (scaled by $10^{2}$).

	Unweighted		MLE		MPLE				CPS
	Treated	Control	Treated	Control	var_set1		var_set2		
					Treated	Control	Treated	Control	
cardiohx	20.4	16.0	18.7	19.3	13.4	14.1	16.3	17.4	NA
chfhx	19.5	16.8	18.5	18.7	13.5	14.1	16.3	16.9	NA
dementhx	6.9	11.6	8.3	9.9	5.5	6.6	6.2	7.9	NA
…		…				…			
race_black	15.3	16.5	16.6	15.8	13.4	12.4	18.6	17.6	12.5
**sex_Female**	41.5	46.1	42.9	44.2	50.3	51.2	50.3	51.6	51.8
age_lt30	4.8	5.1	4.5	4.6	18.4	19.2	6.2	6.2	21.2
age_30_49	18.6	18.4	17.9	18.6	31.6	32.0	23.7	24.6	31.9
age_50_69	40.7	35.9	39.0	38.9	33.3	33.5	51.3	51.9	31.4
**age_ge70**	31.9	36.9	34.4	34.3	14.9	13.9	13.1	12.4	12.6

**TABLE 11:** Estimates of the PATE based on different methods.

Naive	MLE	CBPS	var_set1		var_set2	
			MPLE	SW‐CBPS	MPLE	SW‐CBPS
−0.051 (0.013)	−0.061 (0.017)	−0.062 (0.015)	−0.083 (0.021)	−0.078 (0.019)	−0.08 (0.019)	−0.082 (0.017)

*Note:* The standard errors of the estimates are shown in the parentheses.

## 7 Some Additional Remarks

One of the main messages of the current paper is that appropriate use of survey weights can help mitigate selection bias in causal inference and make the causal statement applicable to the target population. With observational non‐probability samples, our analysis in Section 4 reveals that whether a causal statement is generalizable depends on treatment effect heterogeneity and availability of auxiliary information from external data. The proposed method can be used to assess how sensitive a causal estimate is to selection bias. We focus on Hájek‐type estimators in this paper, while the survey‐weighted inverse propensity score weights can be employed in other contexts such as matching and stratification. These topics might be of interest in future research.

With post‐treatment probability survey samples, we recommend incorporating survey weights in the estimation of the PS because the inclusion of the treatment indicators might be non‐ignorable given the observed confounders. Weighting is not essential if the sampling scheme is conditionally exogenous, that is, the local PS equals the PS. Whether the two PS functions are equal depends on the specific functional form of the inclusion probabilities in relation to design and auxiliary variables. Detailed design information, however, is often not clearly stated in documents for data users or not available in the datasets. The issue of “to weight or not to weight” has been studied previously in the context of regression analysis with survey data. It is believed that if the sampling scheme is conditionally exogenous, then weighting will yield inflated variance of the estimators for regression parameters. Therefore, a preliminary test of whether the sampling scheme is conditionally exogenous was suggested in the literature [46, 47]. However, it is still not clear whether incorporating survey weights in the estimation of the PS will increase or decrease the variance of the treatment effect estimator, and it is difficult to compare them in theory. We recommend that survey weights be incorporated in estimating the PS by the SW‐CBPS method.

For non‐probability samples, the weights for the outcomes are the inverse of the product of the participation probabilities and the PSs. By incorporating the estimated participation probabilities in the estimation of the PS, the influence of extreme values of estimated participation probabilities and their associated variation could be reduced.

Another practical issue is the presence of extreme values of inverse propensity score weights, often arising from limited covariate overlap between the treated and control groups. A typical remedy is to truncate the estimated propensity scores [48]; however, the target subpopulation after truncation is often ill‐defined. A possible solution is to focus on the estimand PATO, which corresponds to the overlap population, a subpopulation of individuals with similar covariates between treatment groups.

## Funding

This work was supported by the Natural Sciences and Engineering Research Council of Canada (RGPIN‐2020‐04345) and the Canadian Statistical Sciences Institute.

## Conflicts of Interest

The authors declare no conflicts of interest.

## Supporting information


**Data S1:** Supporting Information.

## Data Availability

The data that support the findings of this study are openly available in “ATbounds::RHC” in the R package ATbounds at https://search.r‐project.org/CRAN/refmans/ATbounds/html/RHC.html. The R code of our algorithm is available at https://github.com/wliang36/survey‐weighted‐propensity‐score‐weighting.
