# Supplementary material for: Causal Inference With Survey Data: A Robust Framework for Propensity Score Weighting in Probability and Non‐Probability Samples
Source: Stat Med. 2026 Feb 4;45(3-5):e70420. doi: 10.1002/sim.70420 (PMC12873465; doi:10.1002/sim.70420)
Supplement: Supplementary file 1 — Data S1: Supporting Information. [file SIM-45-0-s001.pdf]

Supplementary Materials for "Causal Inference with Survey Data: A Robust Framework for Propensity Score Weighting in Probability and Non-Probability Samples"

Wei Liang and Changbao Wu

1 A Toy Simulated Example

A simple simulated example is given in this section to illustrate the potential biases for estimating the propensity score (PS) and the population weighted average treatment effect (PWATE) when ignoring survey weights in propensity score weighting (PSW) in the presence of a sampling stage.

We generate population data $\{Y_i(1), Y_i(0), X_i, T_i\}_{i=1}^N$ with $N = 1000$ from the following super-population model:

$$
\begin{aligned}
&X \sim \text{Unif}(0, 1),\\
&T \sim \text{Bernoulli}(0.2 + 0.1X),\\
&Y(1) = 1 + 2X + N(0, 0.2^2),\\
&Y(0) = 1 + X + N(0, 0.2^2).
\end{aligned}
$$

Under the above model, the PS function is given by $e(X) = 0.2 + 0.1X$ and the conditional average treatment effect, $\tau(X) = X$, relies on X. We assume that the observed survey samples are drawn using the Poisson sampling method with inclusion probabilities $\pi_i^A = 0.1 + 0.3X_i 1_{X_i \geq 0.5} + 0.05T_i$, which are positively associated with the confounder X_i and depend on T_i conditional on X_i.

We posit a parametric model for the PS as $e(X) = e_{\boldsymbol{\beta}}(X) = \gamma_0 + \gamma_1 X$, and consider the following two different strategies to estimate the PS: (i) maximizing the sample log-likelihood function, termed as maximum sample-likelihood estimation (MSLE), and

Corresponding. Changbao Wu: cbwu@uwaterloo.ca

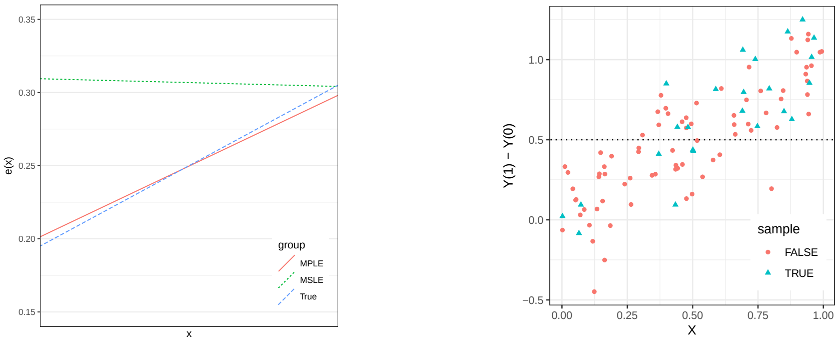

Figure 1: The left plot shows the true PS and the estimated PS functions obtained respectively by MPLE and MSLE based on 2000 Monte Carlo replications. The right plot shows the scatter points of 100 individuals randomly selected from the finite population.

(ii) maximizing the pseudo (survey-weighted) log-likelihood function, termed as maximum pseudo-likelihood estimation (MPLE). The estimated PS functions are plotted in the left panel of Figure 1. We see that the estimated PS based on MSLE, as compared to the one based on MPLE, fails to catch the true PS due to non-ignorable misssingness of T. The right panel of Figure 1 depicts the scatter points of 100 observations, randomly selected from the finite population, with respect to individualized treatment effect $Y(1) - Y(0)$ and the confounder X. Since X_i is positively correlated with both $Y_i(1) - Y_i(0)$ and π_i^A, more individuals with $X_i > 0.5$ are included, and their treatment effects are averagely and comparatively larger than the individuals excluded.

Table 1: Bias (SD) of different Hájek-type weighting estimators (values presented are scaled by 10^2)

Estimand	$\hat{\tau}$	$\hat{\tau}_1$	$\hat{\tau}_2$	$\hat{\tau}_3$
PATE	0.2 (4.1)	5.9 (4.5)	9.1 (4.1)	14.1 (3.1)
PATT	0.8 (5.0)	4.1 (5.3)	9.5 (4.9)	12.1 (4.4)

The biases of the PATE and PATT estimates based on the Hájek-type weighting estimator are given in Table 1. The corresponding standard deviations of the estimates are shown in the parentheses. The $\hat{\tau}$ denotes the SW-IPW estimator where the survey weights are incorporated when estimating the PS and reweighting the outcomes. The $\hat{\tau}_1$ denotes the estimator that ignores the survey weights when estimating the PS. The $\hat{\tau}_2$ denotes the

estimator that ignores the survey weights when reweighting the outcomes. The $\hat{\tau}_3$ denotes the estimator that ignores the survey weights in both steps. We can see that simply incorporating the survey weights in reweighting the outcomes is not sufficient and produced biased estimates. The survey weights are essential in both steps for completely removing the selection bias.

2 Dual Problems of the Survey-Weighted Covariate Balancing Propensity Score Approach

We drive the dual optimization problems to the survey-weighted covariate balancing propensity score (SW-CBPS) approach. The weights constructed based on the SW-CBPS method for the PSATE are the solutions to the optimization problem:

$$\begin{aligned} \min_{\omega_i \geq \tilde{d}_i^A} \sum_{i \in \mathcal{S}_A} (\omega_i - \tilde{d}_i^A) \log(\omega_i - \tilde{d}_i^A) - \omega_i(1 + \log(\tilde{d}_i^A)) \\ \text{s.t.} \sum_{i \in \mathcal{S}_A, T_i=1} \omega_i \boldsymbol{X}_i = \sum_{i \in \mathcal{S}_A, T_i=0} \omega_i \boldsymbol{X}_i. \end{aligned} \tag{1}$$

where $\tilde{d}_i^A = d_i^A 1_{\mathcal{B}}(\boldsymbol{X}_i)$. The objective function of (1) reaches its minimums at $\omega_i = \tilde{d}_i^A$ for $i \in \mathcal{S}_A$. For estimating the PSATT, the weights for the treated groups are $\hat{\omega}_i = \tilde{d}_i^A$ and the estimated weights for the control groups are the solutions to

$$\begin{aligned} \min_{\omega_i > 0} \sum_{i \in \mathcal{S}_A, T_i=0} \omega_i \log(\omega_i / \tilde{d}_i^A) - \omega_i + \tilde{d}_i^A \\ \text{s.t.} \sum_{i \in \mathcal{S}_A, T_i=0} \omega_i \boldsymbol{X}_i = \sum_{i \in \mathcal{S}_A, T_i=1} \tilde{d}_i^A \boldsymbol{X}_i \end{aligned} \tag{2}$$

The objective function of (2) is exactly the Kullback–Leibler divergence which reaches its minimums at $\omega_i = \tilde{d}_i^A$ for $i \in \mathcal{S}_A, T_i = 0$.

The dual problems can obtained based on the technique outlined in Zhao (2019). In the following, we show how to obtain the dual problem for estimating the PSATE. The dual problem for estimating the PSATT can be derived in a similar manner. First, when $g(\boldsymbol{x}) = 1_{\mathcal{B}}(\boldsymbol{x})$, the SW-CBPS method can be formatted equivalently as

$$\begin{aligned} \max_{\boldsymbol{\beta}, \zeta} \sum_{i \in \mathcal{S}_A} \tilde{d}_i^A S(f(\zeta_i), T_i), \\ \text{s.t. } \zeta_i = \boldsymbol{\beta}^\intercal \boldsymbol{X}_i, i \in \mathcal{S}_A, \end{aligned} \tag{3}$$

$f(x) = \text{expit}(x) = \exp(x)/[1+\exp(x)]$ and $S(p,t) = \int (t-p)p^{-2}(1-p)^{-2}dp$. Define the Lagrange function as

$$L(\boldsymbol{\beta}, \boldsymbol{\zeta}, \lambda) = \sum_{i \in \mathcal{S}_A} \tilde{d}_i^A S(f(\zeta_i), T_i) + \sum_{i \in \mathcal{S}_A} \lambda_i(\boldsymbol{\beta}^\intercal \boldsymbol{X}_i - \zeta_i)$$

where $\boldsymbol{\zeta} = (\zeta_1, \cdots, \zeta_N)^\intercal$. The Lagrangian dual problem of (3) is given by $\min_\lambda \max_{\boldsymbol{\beta},\boldsymbol{\zeta}} L(\boldsymbol{\beta}, \boldsymbol{\zeta}, \lambda)$ with the duality gap equal to 0 due to Slater's condition (Boyd et al., 2004). Taking the first derivative of L with respect to $\boldsymbol{\beta}$ and ζ we obtain

$$0 = \frac{\partial L}{\partial \zeta_i} = \tilde{d}_i^A (T_i - f(\zeta_i)) f(\zeta_i)^{-1}(1 - f(\zeta_i))^{-1} - (2T_i - 1)\omega_i, i \in \mathcal{S}_A, \tag{4}$$

$$0 = \frac{\partial L}{\partial \boldsymbol{\beta}} = \sum_{i \in \mathcal{S}_A} (2T_i - 1)\omega_i \boldsymbol{X}_i = 0, \tag{5}$$

where $\omega_i = (2T_i - 1)^{-1}\lambda_i$. Let ω_i be the dual variable of interest and $\tilde{L}(\omega, \boldsymbol{\beta}) = \max_\zeta L(\boldsymbol{\beta}, \zeta, \lambda)$. By solving (4) we get $\zeta_i = T_i f^{-1}(\tilde{d}_i^A/\omega_i) + (1 - T_i) f^{-1}(1 - \tilde{d}_i^A/\omega_i)$ and $\omega_i \geq \tilde{d}_i^A$ for $i \in \mathcal{S}_A$. By substituting ζ_i, some algebra gives

$$\begin{aligned}
\tilde{L}(\omega, \boldsymbol{\beta}) &= \sum_{i \in \mathcal{S}_A} T_i \int \frac{-\omega_i}{\omega_i - \tilde{d}_i^A} d\omega_i - T_i \log\left(\frac{\tilde{d}_i^A}{\omega_i - \tilde{d}_i^A}\right) + (1 - T_i)\log\left(\frac{\omega_i - \tilde{d}_i^A}{\tilde{d}_i^A}\right) \\
&\quad + \sum_{i \in \mathcal{S}_A} (2T_i - 1)\omega_i \boldsymbol{\beta}^\intercal \boldsymbol{X}_i \\
&= \sum_{i \in \mathcal{S}_A} (\omega_i - \tilde{d}_i^A)\log(\omega_i - \tilde{d}_i^A) - \omega_i(1 + \log(\tilde{d}_i^A)) + \sum_{i \in \mathcal{S}_A} (2T_i - 1)\omega_i \boldsymbol{\beta}^\intercal \boldsymbol{X}_i, \omega_i \geq \tilde{d}_i^A.
\end{aligned}$$

The Lagrangian dual problem (1) is given by $\min_\omega \max_{\boldsymbol{\beta}} \tilde{L}(\omega, \boldsymbol{\beta})$.

3 Proof of Main Theorems

3.1 Proof of Theorem 2

Proof. Because $d_i^A = O_p(N/n)$, $e(\boldsymbol{x})$ is strictly bounded between 0 and 1, and $e_{\boldsymbol{\beta}}(\boldsymbol{x})$ is continuous in a neighborhood of $\boldsymbol{\beta}_0$, we have that $\omega_i(\hat{\boldsymbol{\beta}}) = \omega_i(\boldsymbol{\beta}_0) + o_p(N/n)$. It follows that

$$\frac{1}{N}\sum_{i \in \mathcal{S}_A} T_i \hat{\omega}_i = \frac{1}{N}\sum_{i \in \mathcal{S}_A} T_i \omega_i(\boldsymbol{\beta}_0) + o_p(1) = E[g(\boldsymbol{X})] + o_p(1)$$

by the weak law of large numbers (WLLN). Let

$$\varepsilon_i = \frac{g(\boldsymbol{X}_i, \hat{\boldsymbol{\beta}})}{e_{\hat{\boldsymbol{\beta}}}(\boldsymbol{X}_i)} - \frac{g(\boldsymbol{X}_i)}{e(\boldsymbol{X}_i)}$$

where $g(\boldsymbol{X}_i, \hat{\boldsymbol{\beta}})$ denotes the corresponding term of $g(\boldsymbol{X}_i)$ under $\hat{\boldsymbol{\beta}}$ when it is defined in terms of the postulated model for the PS. It is clear that $\varepsilon_i = o_p(1)$. The Cauchy–Schwarz inequality gives

$$b = \frac{1}{N}\sum_{i\in\mathcal{S}_A} T_i(\hat{\omega}_i - \omega(\boldsymbol{\beta}_0))Y_i \leq \left\{\frac{1}{N}\sum_{i\in\mathcal{S}_A} d_i^A T_i Y_i^2\right\}^{1/2}\left\{\frac{1}{N}\sum_{i\in\mathcal{S}_A} d_i^A \varepsilon_i^2\right\}^{1/2}.$$

Because $N^{-1}\sum_{i\in\mathcal{S}_A} d_i^A T_i Y_i^2 = E(Y^2 \mid T = 1) + o_p(1) = O_p(1)$ by the WLLN and $N^{-1}\sum_{i\in\mathcal{S}_A} d_i^A \varepsilon_i^2 = o_p(1)$, we conclude that $b = o_p(1)$. It immediately follows that

$$\frac{1}{N}\sum_{i\in\mathcal{S}_A} T_i\hat{\omega}_i Y_i = \frac{1}{N}\sum_{i\in\mathcal{S}_A} T_i\omega_i(\boldsymbol{\beta}_0)Y_i + b = \frac{1}{N}\sum_{i=1}^{N}\frac{T_i g(\boldsymbol{X}_i)}{e(\boldsymbol{X}_i)}Y_i + o_p(1) = E[g(\boldsymbol{X})Y(1)] + o_p(1)$$

and thus $\hat{\tau}_1 = E[g(\boldsymbol{X})Y(1)]/E[g(\boldsymbol{X})] + o_p(1)$. In a similar manner, we can verify that $\hat{\tau}_0 = E[g(\boldsymbol{X})Y(0)]/E[g(\boldsymbol{X})] + o_p(1)$ and thus $\hat{\tau} = \tau_g + o_p(1)$. □

3.2 Proof of Theorem 3

Proof. Let $\hat{\omega}_i^* = \hat{\omega}_i/\sum_{i\in\mathcal{S}_A} T_i\hat{\omega}_i$ be the normalized weights that satisfy the covariate balancing condition $\sum_{i\in\mathcal{S}_A}(2T_i - 1)\hat{\omega}_i^*\boldsymbol{X}_i = \boldsymbol{0}$. Simple algebra gives that

$$\hat{\tau} - \tau_g = \sum_{i\in\mathcal{S}_A}(2T_i - 1)\hat{\omega}_i^*(Y_i - m_{T_i}(\boldsymbol{X}_i)) + \sum_{i\in\mathcal{S}_A}(2T_i - 1)\hat{\omega}_i^* m_0(\boldsymbol{X}_i) + \sum_{i\in\mathcal{S}_A} T_i\hat{\omega}_i^*\tau(\boldsymbol{X}_i) - \tau_g. \tag{6}$$

The first term at the right side of (6) is negligible because $E[Y_i - m_{T_i}(\boldsymbol{X}_i) \mid \boldsymbol{X}_i, T_i] = 0$. When $m_0(\boldsymbol{X}_i)$ is linear in $\boldsymbol{X}_i$, the second term is zero due to the covariate balancing condition. For estimating the PSATT, we have $T_i\hat{\omega}_i = T_i 1_{\mathcal{B}}(\boldsymbol{X}_i)$. The third term therefore vanishes as

$$\frac{E[\sum_{i\in\mathcal{S}_A} T_i\hat{\omega}_i\tau(\boldsymbol{X}_i)]}{E[\sum_{i\in\mathcal{S}_A} T_i\hat{\omega}_i]} = \frac{E[\sum_{i=1}^{N} e(\boldsymbol{X}_i)1_{\mathcal{B}}(\boldsymbol{X}_i)\tau(\boldsymbol{X}_i)]}{E[\sum_{i=1}^{N} e(\boldsymbol{X}_i)1_{\mathcal{B}}(\boldsymbol{X}_i)]} = \tau_g.$$

For estimating the PSATE and PSATO, the third term is not negligible unless $\tau(\boldsymbol{X}_i)$ is a constant. □

3.3 Proof of Theorem 4

Proof. Recall that $\boldsymbol{Q}(\boldsymbol{\eta}) = E[\boldsymbol{\psi}_{g,\boldsymbol{h}}(\boldsymbol{O}, \boldsymbol{\eta})]$, $\boldsymbol{Q}_N(\boldsymbol{\eta}) = N^{-1}\sum_{i=1}^{N}\boldsymbol{\psi}_{g,\boldsymbol{h}}(\boldsymbol{O}_i, \boldsymbol{\eta})$, and

$$\boldsymbol{Q}_n(\boldsymbol{\eta}) = N^{-1}\sum_{i\in\mathcal{S}_A} d_i^A\boldsymbol{\psi}_{g,\boldsymbol{h}}(\boldsymbol{O}_i, \boldsymbol{\eta}).$$

The $\boldsymbol{\eta}^*$, $\tilde{\boldsymbol{\eta}}$ and $\hat{\boldsymbol{\eta}}$ are respectively the solution to $\boldsymbol{Q}(\boldsymbol{\eta}) = \mathbf{0}$, $\boldsymbol{Q}_N(\boldsymbol{\eta}) = \mathbf{0}$, and $\boldsymbol{Q}_n(\boldsymbol{\eta}) = \mathbf{0}$. In addition, we use $\dot{\boldsymbol{\psi}}_{g,\boldsymbol{h}}$ to denote the first derivative of $\boldsymbol{\psi}_{g,\boldsymbol{h}}$ with respect to $\boldsymbol{\eta}$ (which is a matrix) and let $\boldsymbol{D} = E[\dot{\boldsymbol{\psi}}_{g,\boldsymbol{h}}(\boldsymbol{O}, \boldsymbol{\eta}^*)]$. We impose the following assumptions:

A1 $\boldsymbol{Q}(\boldsymbol{\eta})$, $\boldsymbol{Q}_N(\boldsymbol{\eta})$, and $\boldsymbol{Q}_n(\boldsymbol{\eta})$ are all continuous in $\boldsymbol{\eta}$ and the solutions $\boldsymbol{\eta}^*$, $\tilde{\boldsymbol{\eta}}$ and $\hat{\boldsymbol{\eta}}$ are all unique (almost surely) over $\Theta_{\boldsymbol{\beta}} \times \mathbb{R}^2$.

A2 In a neighborhood of $\boldsymbol{\eta}^*$, $\boldsymbol{\psi}_{g,\boldsymbol{h}}(\boldsymbol{O}, \boldsymbol{\eta})$ is continuously differentiable in $\boldsymbol{\eta}$ almost surely.

A3 Every component of $\boldsymbol{\psi}_{g,\boldsymbol{h}}(\boldsymbol{O}, \boldsymbol{\eta}^*)$ and $\dot{\boldsymbol{\psi}}_{g,\boldsymbol{h}}(\boldsymbol{O}, \boldsymbol{\eta}^*)$ has finite second moment.

A4 The matrix $\boldsymbol{D}$ is positive definite and bounded.

A5 $n^{1/2}\boldsymbol{Q}_n(\tilde{\boldsymbol{\eta}})$ converges in the law of q to $N(0, \boldsymbol{\Omega})$ as $N \to \infty$ where the variance-covariance matrix $\boldsymbol{\Omega}$ is finite and invertible almost surely.

A6 The sampling fraction n/N converges in probability to 0 as $N \to \infty$.

Assumptions **A1** and **A3** guarantee that $\hat{\boldsymbol{\eta}} = \tilde{\boldsymbol{\eta}} + o_p(1)$ and $\tilde{\boldsymbol{\eta}} = \boldsymbol{\eta}^* + o_p(1)$, followed by similar arguments we made in Section 3.1 of the main body. **A2** and **A4** are commonly used smoothness and boundedness assumptions for $\boldsymbol{\psi}_{g,\boldsymbol{h}}$. We now prove that under **A1** - **A6**, we have that $\boldsymbol{\Omega}^{-1/2}\boldsymbol{D}n^{1/2}(\hat{\boldsymbol{\eta}} - \boldsymbol{\eta}^*)$ converges under the joint models to a standard multivariate normal distribution.

Under **A2**, first-order Taylor expansion of $\mathbf{0} = \boldsymbol{Q}_N(\tilde{\boldsymbol{\eta}})$ around $\boldsymbol{\eta}^*$ gives

$$
\begin{aligned}
\mathbf{0} &= \boldsymbol{Q}_N(\boldsymbol{\eta}^*) + \frac{1}{N}\sum_{i=1}^{N} \dot{\boldsymbol{\psi}}_{g,\boldsymbol{h}}(\boldsymbol{O}_i, \boldsymbol{\eta}^*)(\tilde{\boldsymbol{\eta}} - \boldsymbol{\eta}^*) + o_p(\|\tilde{\boldsymbol{\eta}} - \boldsymbol{\eta}^*\|) \\
&= \boldsymbol{Q}_N(\boldsymbol{\eta}^*) + \boldsymbol{D}(\tilde{\boldsymbol{\eta}} - \boldsymbol{\eta}^*) + o_p(\|\tilde{\boldsymbol{\eta}} - \boldsymbol{\eta}^*\|)
\end{aligned}
$$

Under **A3**, $\boldsymbol{Q}_N(\boldsymbol{\eta}^*) = O_p(N^{-1/2})$ by the Chebyshev's inequality. It follows that $N^{1/2}(\tilde{\boldsymbol{\eta}} - \boldsymbol{\eta}^*) = O_p(1)$ under **A4**. Taylor expansion of $\mathbf{0} = \boldsymbol{Q}_n(\hat{\boldsymbol{\eta}})$ about $\tilde{\boldsymbol{\eta}}$ gives

$$
\begin{aligned}
\mathbf{0} &= \boldsymbol{Q}_n(\tilde{\boldsymbol{\eta}}) + \frac{1}{N}\sum_{i \in \mathcal{S}_A} d_i^A \dot{\boldsymbol{\psi}}_{g,\boldsymbol{h}}(\boldsymbol{O}_i, \tilde{\boldsymbol{\eta}})(\hat{\boldsymbol{\eta}} - \tilde{\boldsymbol{\eta}}) + o_p(\|\hat{\boldsymbol{\eta}} - \tilde{\boldsymbol{\eta}}\|) \\
&= \boldsymbol{Q}_n(\tilde{\boldsymbol{\eta}}) + \frac{1}{N}\sum_{i \in \mathcal{S}_A} d_i^A \dot{\boldsymbol{\psi}}_{g,\boldsymbol{h}}(\boldsymbol{O}_i, \boldsymbol{\eta}^*)(\hat{\boldsymbol{\eta}} - \tilde{\boldsymbol{\eta}}) + o_p(\|\hat{\boldsymbol{\eta}} - \tilde{\boldsymbol{\eta}}\|) \\
&= \boldsymbol{Q}_n(\tilde{\boldsymbol{\eta}}) + \boldsymbol{D}(\hat{\boldsymbol{\eta}} - \tilde{\boldsymbol{\eta}}) + o_p(\|\hat{\boldsymbol{\eta}} - \tilde{\boldsymbol{\eta}}\|)
\end{aligned}
$$

Because $n^{1/2}\boldsymbol{Q}_n(\tilde{\boldsymbol{\eta}}) = O_p(1)$ under **A5**, we immediately conclude that $\hat{\boldsymbol{\eta}} - \tilde{\boldsymbol{\eta}} = O_p(n^{-1/2})$ under **A4**. More specifically, we have

$$\boldsymbol{\Omega}^{-1/2}\boldsymbol{D}n^{1/2}(\hat{\boldsymbol{\eta}} - \tilde{\boldsymbol{\eta}}) = \boldsymbol{\Omega}^{-1/2}n^{1/2}\boldsymbol{Q}_n(\tilde{\boldsymbol{\eta}}) + o_p(1).$$

Based on the above results and **A6**, we obtain

$$\begin{aligned}\boldsymbol{\Omega}^{-1/2}\boldsymbol{D}n^{1/2}(\hat{\boldsymbol{\eta}} - \boldsymbol{\eta}^*) &= \boldsymbol{\Omega}^{-1/2}\boldsymbol{D}n^{1/2}(\hat{\boldsymbol{\eta}} - \tilde{\boldsymbol{\eta}}) + (n/N)^{1/2}\boldsymbol{\Omega}^{-1/2}\boldsymbol{D}N^{1/2}(\tilde{\boldsymbol{\eta}} - \hat{\boldsymbol{\eta}}) \\ &= \boldsymbol{\Omega}^{-1/2}n^{1/2}\boldsymbol{Q}_n(\tilde{\boldsymbol{\eta}}) + o_p(1).\end{aligned}$$

Since $n^{1/2}\boldsymbol{\Omega}^{-1/2}\boldsymbol{Q}_n(\tilde{\boldsymbol{\eta}})$ converges in the law of q to $N(0, I)$, it also converges under the joint models to $N(0, I)$ by the bounded convergence theorem. Therefore, $\boldsymbol{\Omega}^{-1/2}\boldsymbol{D}n^{1/2}(\hat{\boldsymbol{\eta}} - \boldsymbol{\eta}^*)$ converges under the joint models to $N(0, I)$ by the Slutsky's theorem. This completes the proof. □

3.4 Proof of Theorem 6

Proof. Under **C2**, we can write $g(\boldsymbol{X}) = g(\boldsymbol{X}_{\mathcal{C}})$. Define $m_{l,t} = E(Y \mid R = 1, T = t, \boldsymbol{X})$ for $t = 0, 1$. Some algebra gives that

$$\begin{aligned}&E\{T_i\omega_i(\boldsymbol{\theta}_0, \boldsymbol{\beta}_0)Y_i\} - E\{(1 - T_i)\omega_i(\boldsymbol{\theta}_0, \boldsymbol{\beta}_0)Y_i\} \\ =&E\left\{\frac{RTg(\boldsymbol{X})}{\pi(\boldsymbol{X}_{\mathcal{C}})e(\boldsymbol{X})}Y\right\} - E\left\{\frac{R(1-T)g(\boldsymbol{X})}{\pi(\boldsymbol{X}_{\mathcal{C}})[1 - e(\boldsymbol{X})]}Y\right\} \\ =&E\left\{\frac{RTg(\boldsymbol{X})}{\pi(\boldsymbol{X}_{\mathcal{C}})e(\boldsymbol{X})}m_{l,1}(\boldsymbol{X})\right\} - E\left\{\frac{R(1-T)g(\boldsymbol{X})}{\pi(\boldsymbol{X}_{\mathcal{C}})[1 - e(\boldsymbol{X})]}m_{l,0}(\boldsymbol{X})\right\} \\ =&E\left\{\frac{Rg(\boldsymbol{X})}{\pi(\boldsymbol{X}_{\mathcal{C}})}[m_{l,1}(\boldsymbol{X}) - m_{l,0}(\boldsymbol{X})]\right\} && \text{under } \mathbf{C1} \\ =&E\left\{\frac{Rg(\boldsymbol{X}_{\mathcal{C}})}{\pi(\boldsymbol{X}_{\mathcal{C}})}\tau(\boldsymbol{X})\right\} && \text{under } \mathbf{C2} \text{ and } \mathbf{C3} \\ =&E\{g(\boldsymbol{X}_{\mathcal{C}})\tau(\boldsymbol{X})\}. && \text{under } \mathbf{C4}\end{aligned}$$

This proof is completed by noting that $E\{T_i\omega_i(\boldsymbol{\theta}_0, \boldsymbol{\beta}_0)\} = E\{(1 - T_i)\omega_i(\boldsymbol{\theta}_0, \boldsymbol{\beta}_0)\} = E[g(\boldsymbol{X})]$. □

3.5 Proof of Theorem 8

Proof. Some algebra gives that

$$
\begin{aligned}
& E\left\{T_i\omega_i(\boldsymbol{\theta}_0,\boldsymbol{\beta}_0)Y_i\right\} - E\left\{(1-T_i)\omega_i(\boldsymbol{\theta}_0,\boldsymbol{\beta}_0)Y_i\right\} \\
=& E\left\{\frac{RTg(\boldsymbol{X})}{\pi(\boldsymbol{X}_{\mathcal{C}})e_l(\boldsymbol{X})}Y(1)\right\} - E\left\{\frac{R(1-T)g(\boldsymbol{X})}{\pi(\boldsymbol{X}_{\mathcal{C}})[1-e_l(\boldsymbol{X})]}Y(0)\right\} \\
=& E\left\{\frac{Rg(\boldsymbol{X})}{\pi(\boldsymbol{X}_{\mathcal{C}})}Y(1)\right\} - E\left\{\frac{Rg(\boldsymbol{X})}{\pi(\boldsymbol{X}_{\mathcal{C}})}Y(0)\right\} && \text{under local ignorability} \\
=& E\left\{\frac{Rg(\boldsymbol{X})}{\pi(\boldsymbol{X}_{\mathcal{C}})}[Y(1)-Y(0)]\right\} \\
=& E\left\{\frac{Rg(\boldsymbol{X}_{\mathcal{C}})}{\pi(\boldsymbol{X}_{\mathcal{C}})}[Y(1)-Y(0)]\right\} && \text{under } \textbf{C2} \\
=& E\left\{g(\boldsymbol{X}_{\mathcal{C}})[Y(1)-Y(0)]\right\}. && \text{under either } \textbf{E4} \text{ or that } \textbf{E3} \text{ and } \textbf{C4}
\end{aligned}
$$

The proof is completed. □

References

Boyd, S., Boyd, S. P., and Vandenberghe, L. (2004), *Convex Optimization*, Cambridge: Cambridge University Press.

Zhao, Q. (2019), "Covariate balancing propensity score by tailored loss functions," *The Annals of Statistics*, 47, 965–993.
